# Gene Expression Reaction Norms Unravel the Molecular and Cellular Processes Underpinning the Plastic Phenotypes of *Alternanthera Philoxeroides* in Contrasting Hydrological Conditions

**DOI:** 10.3389/fpls.2015.00991

**Published:** 2015-11-12

**Authors:** Lexuan Gao, Yupeng Geng, Hongxing Yang, Yonghong Hu, Ji Yang

**Affiliations:** ^1^Center for Evolutionary Biology and Institute of Biodiversity Science, Fudan UniversityShanghai, China; ^2^Shanghai Key Laboratory of Plant Functional Genomics and Resources, Shanghai Chenshan Plant Science Research Center, Chinese Academy of Sciences, Shanghai Chenshan Botanical GardenShanghai, China; ^3^School of Ecology and Environmental Sciences, Institute of Ecology and Geobotany, Yunnan UniversityKunming, China

**Keywords:** *Alternanthera philoxeroides*, phenotypic plasticity, transcriptional plasticity, genomic reaction norm, molecular processes

## Abstract

*Alternanthera philoxeroides* is an amphibious invasive weed that can colonize both aquatic and terrestrial habitats. Individuals growing in different habitats exhibit extensive phenotypic variation but little genetic differentiation. Little is known about the molecular basis underlying environment-induced phenotypic changes. Variation in transcript abundance in *A. philoxeroides* was characterized throughout the time-courses of pond and upland treatments using RNA-Sequencing. Seven thousand eight hundred and five genes demonstrated variable expression in response to different treatments, forming 11 transcriptionally coordinated gene groups. Functional enrichment analysis of plastically expressed genes revealed pathway changes in hormone-mediated signaling, osmotic adjustment, cell wall remodeling, and programmed cell death, providing a mechanistic understanding of the biological processes underlying the phenotypic changes in *A. philoxeroides*. Both transcriptional modulation of environmentally sensitive loci and environmentally dependent control of regulatory loci influenced the plastic responses to the environment. Phenotypic responses and gene expression patterns to contrasting hydrological conditions were compared between *A. philoxeroides* and its alien congener *Alternanthera pungens*. The terricolous *A. pungens* displayed limited phenotypic plasticity to different treatments. It was postulated based on gene expression comparison that the interspecific variation in plasticity between *A. philoxeroides* and *A. pungens* was not due to environmentally-mediated changes in hormone levels but to variations in the type and relative abundance of different signal transducers and receptors expressed in the target tissue.

## Introduction

Phenotypic plasticity, the ability of one genotype to produce different phenotypes in response to different environments (Bradshaw, 1965; Schlichting, 1986; Scheiner, 1993; Pigliucci and Hayden, 2001; Pigliucci, 2005; Zhou et al., 2012), is widely recognized as a rapid adaptive strategy that enables plants to survive in different habitats (Daehler, 2003; Hulme, 2008; Prentis et al., 2008; Sultan et al., 2013). Plastic responses to environmental variation allow organisms to produce advantageous phenotypes in heterogeneous habitats without the time cost required for natural selection, thus allow individuals to withstand the challenges of environmental variability and increase their niche breadth (Bradshaw, 1965; Sultan et al., 1998, 2009, 2013; Donohue et al., 2001; Richards et al., 2005; Sultan, 2010). Successful invasive species are usually characterized by broader niche breadth and greater environmental tolerance (Williamson, 1996; Vazquez, 2006; Higgins and Richardson, 2014). Phenotypic plasticity has long been proposed to play an important role in facilitating successful invasions (Daehler, 2003; Richards et al., 2006; Hulme, 2008; Prentis et al., 2008; Knop and Reusser, 2012; Sultan et al., 2013; Morris et al., 2014). Understanding how adaptive plasticity occur in changing environments can not only illuminate the role of phenotypic plasticity in shaping the niche breath, but may also help account for the success of invasive species (Davidson et al., 2011; Grether, 2014).

*Alternanthera philoxeroides*, commonly known as alligator weed, is an invasive amphibious weed that is native to South America but has now invaded into the temperate and tropical regions across the world (Julien et al., 1995; Holm et al., 1997; Pan et al., 2007). In its introduced range, *A. philoxeroides* rarely produces viable seeds and propagates mainly via vegetative regeneration, with fragments of stems or roots breaking off and growing into new plants (Julien and Stanley, 1999; Geng et al., 2007; Pan et al., 2007). All new plants produced in this way are genetically identical clones. Interestingly, these clones can exploit extremely diverse habitats, from lakes to dry lands (Huai et al., 2003; Pan et al., 2007). Individuals growing in different habitats exhibited notable morphological differences (Geng et al., 2007; Gao et al., 2010). It has therefore been suggested that phenotypic plasticity rather than the development of locally adapted ecotypes is responsible for this species' ability to colonize a wide range of habitats with very different levels of water availability (Geng et al., 2006, 2007; Li and Ye, 2006; Pan et al., 2007).

Although encompassing various phenomena spanning multiple levels of organization, most phenotypically plastic responses seem to take place by altering gene expression and eventually altering ontogenetic trajectory in response to environmental variation (Schmitt et al., 1999; Aubin-Horth and Renn, 2009; Roelofs et al., 2010; Matsumoto and Crews, 2012; Renn and Schumer, 2013). How environmental cues triggering plastic responses at the molecular level and subsequently inducing phenotypic plastic changes in organisms living in fluctuating environments, has been a key focus in ecological developmental biology (Sultan, 2010). With the development of next-generation sequencing technologies, many studies have been conducted to investigate environment-gene interactions at the whole genome level (Landry et al., 2006; Stern et al., 2007; Richards et al., 2012; Zhou et al., 2012; Johansson et al., 2013; Smith et al., 2013a,b; Morris et al., 2014). Transcriptome-wide expression variations associated with environmental fluctuation have been detected in various organisms (Dal Santo et al., 2013; Smith et al., 2013a,b; van Veen et al., 2013). By incorporating functional annotation of differentially expressed genes, great progress has been made in our understanding of the signaling pathways and molecular processes involved in environment-induced phenotypic changes (Aubin-Horth and Renn, 2009).

Characterizing environment-specific gene expression is important not only for elucidating the molecular mechanisms underlying phenotypic plasticity, but also for identifying candidate genes potentially responsible for the plasticity (Bar-Joseph et al., 2012; Romero et al., 2012). However, simply correlating phenotypes with gene expression patterns across environments is insufficient for establishing a causal link between environmental cue, gene expression and the resulting phenotype, because gene expression is itself a plastic trait that can be the result of responses to environmental fluctuations (Côté et al., 2007). To determine whether the observed differential expression is a cause of the plastic change in phenotype or a consequence associated with the new phenotype, it is important to track the progression of transcriptional variation across the developmental time-course of phenotypic changes, but not to focus on the expression patterns associated with the final phenotypes (Aubin-Horth and Renn, 2009; Bar-Joseph et al., 2012). Genes that trigger plastic development may only be differentially expressed during the initial transition period when developmental change is initiated (Aubin-Horth and Renn, 2009). It is also clear that gene expression itself is subject to both genetic variation and environmental changes (Bossdorf et al., 2008). Transcriptome variation across environments may be genetically based differences between individuals rather than inducible differences (plasticity). It has proven difficult to conclusively distinguish the relative contribution of genetic variants and environmental perturbations to gene expression variation in sexually reproducing organisms (Gibson, 2008; Montgomery and Dermitzakis, 2009). *A. philoxeroides* seems to provide a suitable model for explicitly addressing the environmental effects on gene expression variation in an ecological context. Due to its clonal propagation, the genetic variability that inevitably accompanies sexual reproduction is largely eliminated in *A. philoxeroides*.

Previously, we have conducted an extensive investigation of the phenotypic variation of *A. philoxeroides* in natural populations. Using a common-garden experimental protocol, we have also documented the growth reaction norms of *A. philoxeroides* in response to contrasting hydrological conditions (Geng et al., 2006, 2007; Gao et al., 2010). However, little is known about the molecular basis underlying the environment-induced phenotypic changes in this species. In this study, we examined the time-course of gene expression changes induced by contrasting hydrological conditions in *A. philoxeroides* using high-throughput RNA-Sequencing (RNA-Seq). The reference transcriptome of *A. philoxeroides* was generated by *de novo* assembly. Temporally dynamic changes in gene expression was assessed by digital gene expression profiling. Functional annotation of differentially expressed genes will provide information for identifying the core transcriptional responses to environmental variations and the molecular basis underlying the development of plastic traits.

*Alternanthera pungens* is an alien congener of *A. philoxeroides*. In contrast to the wide distribution of *A. philoxeroides* in various aquatic and terrestrial habitats in southern China, *A. pungens* is limitedly distributed in the terrestrial habitats in Yunnan, Fujian and Hainan provinces. It has also been shown that *A. pungens* exhibited much less phenotypic variation in internode length, shoot diameter and pith cavity diameter than the plants of *A. philoxeroides* in response to changing hydrological conditions (Wang et al., 2010). The interspecific difference in phenotypic plasticity could be one of the factors contributing to the niche breadth and geographic variations between *A. philoxeroides* and *A. pungens*. The objectives of this study were: (1) to investigate the molecular underpinnings underlying the environment-induced phenotypic variation in *A. philoxeroides* by monitoring the change in gene expression patterns over time; (2) to identify molecular determinants potentially responsible for variation in plasticity between *A. philoxeroides* and *A. pungens* based on gene expression comparison.

## Materials and methods

### Plant materials and common garden treatments

Plants of *Alternanthera philoxeroides* (Mart.) Griseb. were collected in Zhuji, Zhejiang Province (E120°20′, N29°40′) in 2006. The collected materials were grown in nutrient soil and had been maintained in a greenhouse in Fudan University, Shanghai (E121°29′, N31°14′) for more than 5 years. Stem fragments of one individual plant were cut from ramets with similar diameters and planted in plastic plates. After the first two new leaves appeared, plants of similar sizes were individually transplanted into sand pots, and were watered every day to keep the matrix moist. *A. pungens* was collected from Yunnan, China. Seeds were pre-germinated for 3 days, then the seedlings were individually transplanted into sand pots and grown under the same conditions as *A. philoxeroides*.

Two common garden treatments, pond and upland treatments, were established in the greenhouse to simulate the aquatic and terrestrial habitats colonized by *A. philoxeroides* in natural environments. Two treatments were conducted simultaneously, during the summer months of July and August with about 12 h of daytime, and under identical climatic conditions. In the pond treatment, the potted plants were completely submerged in a series of plastic tanks containing tap water to a depth of 50 cm, three or four plants per tank. Plants were supplied with 1 L water per day in the upland treatment and the soil was kept well-drained. One-month-old plants of uniform size were selected for treatments. Eighty plants were randomly assigned into the upland and pond treatments. Each treatment contained 40 plants, which were assigned into two blocks randomly. Twenty plants were located randomly in each block. In each treatment, ten plants were used for monitoring the changing trends in stem internode length and stem pitch cavity diameter, five plants were used for anatomical observation and monitoring cell death associated with pith cavity formation, five plants were used for RNA extraction for *de novo* transcriptome assembly, and twenty plants were used for temporal expression profile analysis. Plants of *A. pungens* were subjected to similar treatments under the same conditions.

### Phenotypic evaluation

Previous studies have shown that plants of *A. philoxeroides* growing in different habitats exhibited significant phenotypic differences. Particularly, plants in aquatic habitats had significantly longer internodes and larger stem pith cavity than those in terrestrial habitats (Geng et al., 2007; Gao et al., 2010). These two traits were thus chosen as markers of phenotypic variation under different conditions in this study. Phenotypic observations were focused on fresh mature stem internodes between the fifth and sixth nodes. The stem internode length and stem pitch cavity diameter under upland and pond treatments were measured daily on days 0–3 from the start of the treatments. Ten branches from separate plants were randomly collected from each treatment. To monitor cell death associated with pith cavity formation, five branches of separate plants subjected to 48 h submergence were randomly collected and were stained with 2% Evans blue (Gaff and Okong'o-Ogola, 1971) for 3 min, and then washed in water for 2 h before inspection. For anatomical observation, plants were grown under the appropriate conditions for 15 days. Then, five branches of separate plants were randomly collected and used for paraffin sectioning and observation using a light microscope in each treatment.

### RNA-Seq analysis of the genome-wide transcription dynamics of *A. philoxeroides* in response to changing hydrological conditions

Transcriptome sequencing and *de novo* assembly were used to create a reference sequence resource for *A. philoxeroides*. After 1 month of treatment, tender shoots, mature leaves, young stems, and mature stems were harvested from plants in the pond and upland treatment groups. Five replicates from separate plants were collected for each tissue and treatment. All samples were stored in RNAlater (Ambion) until use. Total RNA was isolated using TRIzol Reagent (Invitrogen), following the manufacturer's protocol. The quality and quantity of obtained RNA were evaluated on a Bioanalyzer 2100 (Agilent Technologies). To obtain comprehensive data on expressed gene sequences, RNA samples from both treatments and four separate tissues of five replicates were pooled and used for library construction.

cDNA library construction and Illumina pair-end sequencing were performed at Beijing Genomics Institute (BGI), Shenzhen, China (http://www.genomics.cn/index.php) according to instructions provided by Illumina Inc. The sequence data were deposited in the US National Center for Biotechnology Information (NCBI) Sequence Read Archive (SRA, http://www.ncbi.nlm.nih.gov/Traces/sra; Wheeler et al., 2008) under accession number SRP044887.

*De novo* transcriptome assembly was performed using SOAPdenovo v1.04 with default settings (Li et al., 2010). The BLASTx program was used to align the assembled unigenes against the NCBI non-redundant protein (Nr) database (http://www.ncbi.nlm.nih.gov) and the *Arabidopsis* protein database at The Arabidopsis Information Resource (TAIR, http://www.arabidopsis.org) with an *E*-value threshold of 10^−10^ at a 30% identity. Functional annotation by Gene Ontology (GO, http://www.geneontology.org) terms were obtained using the Blast2GO program (Conesa et al., 2005). In addition, we obtained metabolic pathway annotations for each hit by searching against the Kyoto Encyclopedia of Genes and Genomes (KEGG, http://www.genome.jp/kegg) pathway database (Ogata et al., 1999) using the BLASTx program with an *E*-value cutoff of 10^−10^.

To establish a temporal map of gene expression in *A. philoxeroides*, stem internode tissues were harvested at 0, 1, 3, 6, 12, 24, 48, 120, and 288 h from the pond and upland treatments. Seven replicates from separate plants were collected for each time point and treatment. The internodes were selected for sampling because they exhibit significant phenotypic plasticity under different water conditions with respect to multiple traits including pith cavity size and internode length (Gao et al., 2010). Total RNA was isolated using TRIzol Reagent (Invitrogen). RNAs of the seven replicates for each sample were pooled and used for library construction. Differential single-ended gene expression libraries were constructed using TruSeq RNA Sample Preparation Kit (Illumina) according to the manufacturer's instructions and sequenced using an Illumina HiSeq 2000 system. The sequence data are available at the NCBI SRA with the accession number SRP044889.

After Illumina sequencing, the raw reads were processed to remove adaptors, low-quality reads and reads with unknown bases. The remaining high quality reads (clean reads) for each sample were separately aligned to our reference transcriptome using SOAPaligner/soap2 (Li et al., 2009); 1 bp mismatches were allowed. Gene expression levels were calculated from the number of uniquely aligned clean reads and then normalized into units of Reads Per Kilobase per Million reads mapped (RPKM; Mortazavi et al., 2008). A previously described method (Audic and Claverie, 1997) was used to identify differentially expressed genes. The false discovery rate (FDR) control method was adopted to correct *P*-values in multiple hypothesis tests. Fold changes in gene expression were determined from RPKM ratios. A gene was considered to be differentially expressed if it had an FDR ≤ 0.001 and the absolute value of log_2_Ratio ≥ 1.

Differentially expressed genes were clustered and visualized using the Self-Organizing Map (SOM) algorithm (Kohonen, 1982) based on the similarity of expression patterns. SOM clustering was initially applied to histogram-normalized log_2_Ratio data. Seven thousand eight hundred and five differentially expressed genes were assigned to 442 hexagonal SOM units. Differentially expressed genes sharing similar expression patterns were assigned to the same hexagonal SOM unit, and the hexagonal SOM units with similar average expression patterns were adjacent to each other in the component planes. The resulting hexagonal SOM units were then grouped by *k*-means clustering (*k* = 11) in order to identify SOM regions corresponding to coherent expression patterns. More coherent hexagonal SOM units were identified by applying a Euclidian distance coefficient threshold of 0.3 and then generated SOM clusters. Finally, based on the median expression pattern of each SOM cluster, which was calculated from the median values of log_2_Ratio of all genes at each time point, the best-matched 50% of all genes within each SOM cluster were plotted to visualize gene expression variation patterns. GO enrichment analysis was performed on the gene groups obtained by SOM clustering using the R Bioconductor topGO package with default arguments (Gentleman et al., 2004; Alexa et al., 2006). Fisher's exact test was used to determine significance of enrichment.

To validate the reliability of the gene expression data obtained by RNA-Seq, the expression levels of genes of interest were measured by real-time quantitative reverse transcription polymerase chain reaction (qRT-PCR) under pond and upland conditions at different time points. First strand cDNA was synthesized using the PrimeScript RT (Perfect Real Time) kit (TAKARA). The correctness of the gene sequences obtained from the reference transcriptome was verified by reverse transcription PCR using gene-specific primers, followed by TA cloning using PMD19-T vector kit (TAKARA) and sequencing. qRT-PCR was performed on a Roche Real-time PCR System (LightCycler 480) using SYBR Green PCR Master Mix (TAKARA). Three independent biological replicates were performed for each reaction. The gene-specific primers used for reverse transcription PCR and qRT-PCR (Supplementary Table 1) were designed using PRIMERS3 (Koressaar and Remm, 2007; Untergasser et al., 2012). The Illumina sequencing data revealed a stably expressed gene homologous to *Arabidopsis* ubiquitin-conjugating enzyme 10 (*UBC10*); this gene was used as internal reference. Relative expression levels of target genes were calculated using the 2^−ΔΔ^^Ct^ method (Livak and Schmittgen, 2001).

### Experimental validation and comparative analysis between *A. philoxeroides* and *A. pungens*

Different methods were used to verify the key molecular and cellular processes revealed by gene expression analysis that underpin the plastic phenotypes of *A. philoxeroides*. Comparative analyses between *A. philoxeroides* and *A. pungens* were conducted to detect molecular determinants potentially responsible for variation in plasticity between two species.

To evaluate degrees of phenotypic variance via plasticity to different water availability in *A. philoxeroides* and *A. pungens*, plants of both species were subjected to pond and upland treatments for 15 days before determining the stem internode elongation length. The measurement was conducted randomly on 15 branches from separate plants for each treatment and species.

To verify the role of endogenous hormones in mediating plastic gene expression and phenotypes cued by the external environment, treatments with exogenous ethylene and gibberellin (GA) and their inhibitors were carried out in common garden experiments. The ethylene treatment was performed by placing the potted plants in closed chambers with atmosphere containing 10 ppm ethylene under the upland condition, three plants per chamber. In the case of GA treatment under the upland condition, the potted plants were treated with GA_3_ aqueous solutions at a concentration of 10 μM. Plants were treated by leaf spraying and root watering once a day. Submerged GA-treated plants were flooded in 10 μM of GA_3_. In addition, separate groups of plants were pretreated with the ethylene perception inhibitor 1-MCP (10 ppm) or the GA biosynthesis inhibitor uniconazole (1.5 μM) for 4 days before the experiments. Plants of both species were subjected to each treatment for 10 days before determining the internode elongation length, the measurement was conducted randomly on 15 branches from separate plants for each treatment and species. For both species, stem internode tissues were collected at 6, 24, 48, and 72 h from the ethylene, GA, pond and upland treatments for RNA extraction. For each species, six replicates from separate plants were collected for each time point and treatment.

To monitor the changing trend in cellular osmotic potential under submergence in both species, cell saps of submerged plants were collected from stem segments at 0, 1, 3, 6, 9, and 12 h of the pond treatment. Osmotic properties were determined using a PSYPRO system (Wescor). Eight branches from separate plants of each species were randomly collected for each time point for measuring osmotic potential. Meanwhile, stem internode tissues were collected for both species at 1, 3, 6, 9, and 12 h of the pond and upland treatments for RNA extraction. Six replicates from separate plants were collected for each time point and treatment.

The expression levels of genes encoding α-expansins and three genes homologous to *Arabidopsis ESKIMO 1* (*ESK1*, AT3G55990), *cellulose synthase A8* (*CESA8*, AT4G18780) and *methionine synthase 1* (*MS1*, AT5G17920), respectively, were analyzed by qRT-PCR in both species. The *A. pungens* sequences of these genes were obtained by reverse transcription PCR using the primers designed based on the sequences of *A. philoxeroides* (Supplementary Table 1). PCR products were cloned using the PMD19-T vector kit (TAKARA). Ten clones were randomly selected and sequenced for each PCR product. Three independent biological replicates were performed for each qRT-PCR reaction. The expansin genes are downstream targets of the signal transduction pathway that induce cell wall loosening and ultimately facilitate elongation (Cosgrove, 2000; Lee et al., 2001). Arabidopsis *ESK1, CESA8*, and *MS1* have been predicted to be involved in osmotic stress response (Bohnert and Jensen, 1996; Narita et al., 2004; Chen et al., 2005; Bouchabke-Coussa et al., 2008).

## Results

### Phenotypically plastic variation of *A. philoxeroides* under different treatments

**Figure 1:**
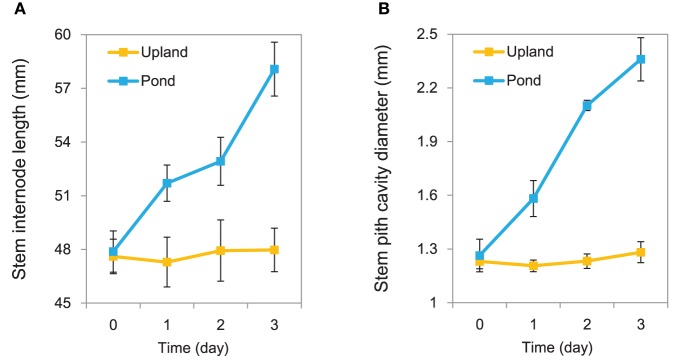
**Phenotypic variation of *Alternanthera philoxeroides* under upland and pond conditions**. **(A)** Variation trends in stem internode elongation. **(B)** Variation trends in stem pith cavity enlargement. Quoted values are means + *s.d., n* = 10.

Growth in the pond condition promoted rapid elongation of stem internodes in *A. philoxeroides* (Figure 1A). The internode cells were clearly wider (based on transverse sections) and longer (based on longitudinal sections) for plants grown in the pond condition than those in the upland condition (Supplementary Figure 1). In addition, *A. philoxeroides* constitutively forms gas spaces in its stems that are known as pith cavity. Growth under pond conditions caused further development of the pith cavity (Figure 1B). Evans blue staining indicated the formation of lysigenous aerenchyma during the extension of pith cavity (Supplementary Figure 2). The extent of cell death increased after 48 h of growth in the pond condition (Supplementary Figure 2).

### *De novo* assembly and annotation of *A. philoxeroides* transcriptome

*De novo* transcriptome assembly using ~55.1 million clean reads generated 144,082 unigenes (Supplementary Table 2). Of them, 10,242 were greater than 1000 bp in length (Supplementary Table 3). 39,055 (27.11%) showed significant similarity to known proteins in the NCBI Nr database and the TAIR database. 35,890 unigenes were assigned to 96 GO categories (Supplementary Figure 3). KEGG pathway analysis showed that 34,400 (23.88%) unigenes showed significant similarity to the known proteins in the KEGG database, and 16,107 could be mapped to 125 pathways (Supplementary Table 4).

### Expression profiling of *A. philoxeroides* in contrasting hydrological conditions

**Figure 2:**
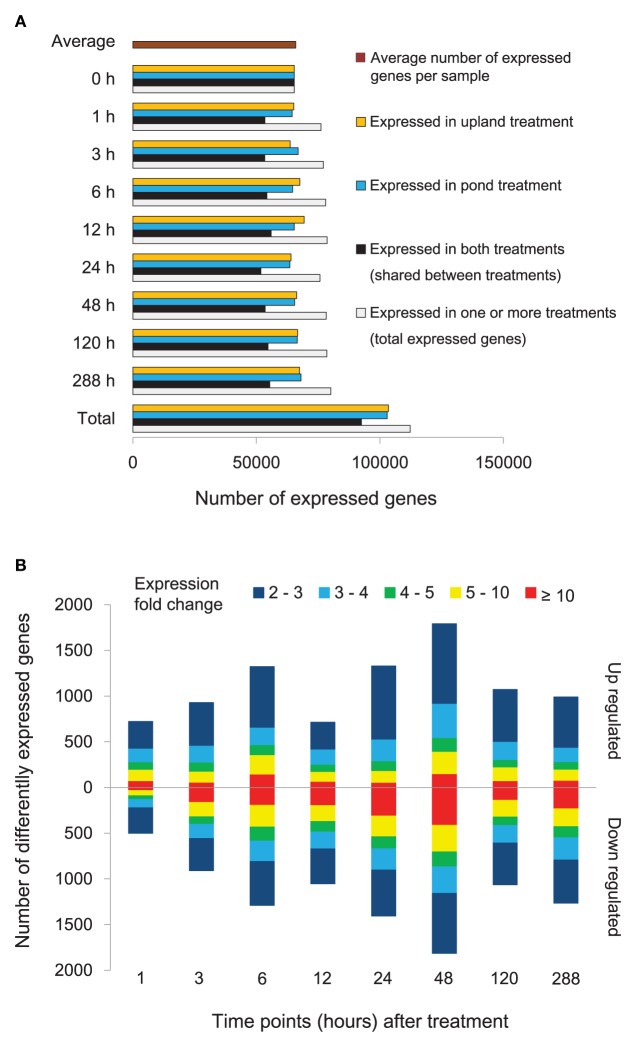
**Gene expression of *Alternanthera philoxeroides* in contrasting hydrological habitats**. **(A)** Number of unigenes expressed at different time points under the pond and upland treatments. **(B)** Genes exhibiting differential expression between the pond and upland treatments at different time points. Bars pointing up and down indicate the number of genes whose expression was up- or down-regulated in the pond treatment relative to the upland treatment, respectively. Fold changes in expression are color-coded.

Temporal patterns of gene expression in *A. philoxeroides* grown in contrasting hydrological conditions were obtained by time series transcriptome analyses using RNA-Seq. Seventeen independent cDNA libraries were generated and sequenced. A total of 123.12 million single-end clean reads of 49 bp in length were obtained. On average, 3.95 million reads per library were aligned to unique location in our *A. philoxeroides* reference transcriptome (Supplementary Table 5). In total, 134,784 (93.54%) unigenes with at least one uniquely mapped read were detected in at least one sample. To minimize false positives for expressed genes, at least two uniquely mapped reads were required for detectable expression of a given unigene. The number of expressed unigenes for different time points and treatments is shown in Figure 2A. In total, 112,320 (77.96%) unigenes were expressed in at least one sample, and the average number of expressed unigenes per sample was 65,991. By comparing the gene expression profiles for the pond and upland treatments at each time point, we identified 7805 differentially expressed genes, including 1589 genes whose intensity of expression differed more than 10-fold (Figure 2B, Supplementary Table 6). In addition, there were 1266 genes that exhibited differential expression within 1 h of the start of treatment (Figure 2B). The expression profiling data obtained by RNA-Seq was verified by qRT-PCR. The transcript abundances of 17 genes of interest measured by RNA-Seq at different time points of the treatments showed a significant correlation with the qRT-PCR measurements (*R* = 0.893, *P* = 5.04*E*-20; Supplementary Figure 4).

**Figure 3:**
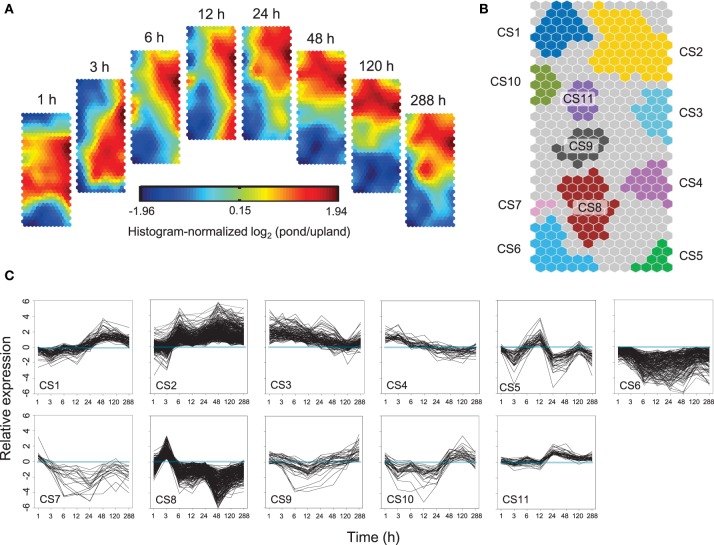
**Temporal patterns of gene expression in *Alternanthera philoxeroides* in contrasting hydrological habitats**. **(A)** Component planes of a Self-Organizing Maps (SOM) fitted to the treatment time point data set. Each component plane shows histogram-normalized variation in gene expression [log_2_ (pond/upland)] at one time point, using a color gradient from blue to red to indicate up- and down-regulation (see color bar). **(B)** Eleven robust clusters (CS1–CS11) were identified; the separate clusters are color-coded. **(C)** Temporal expression profiles for the 11 clusters are plotted based on the 50% of best-fitting genes in each. Time points are plotted on the X-axis while the Y-axis indicates the value of log_2_ (pond/upland).

**Figure 4:**
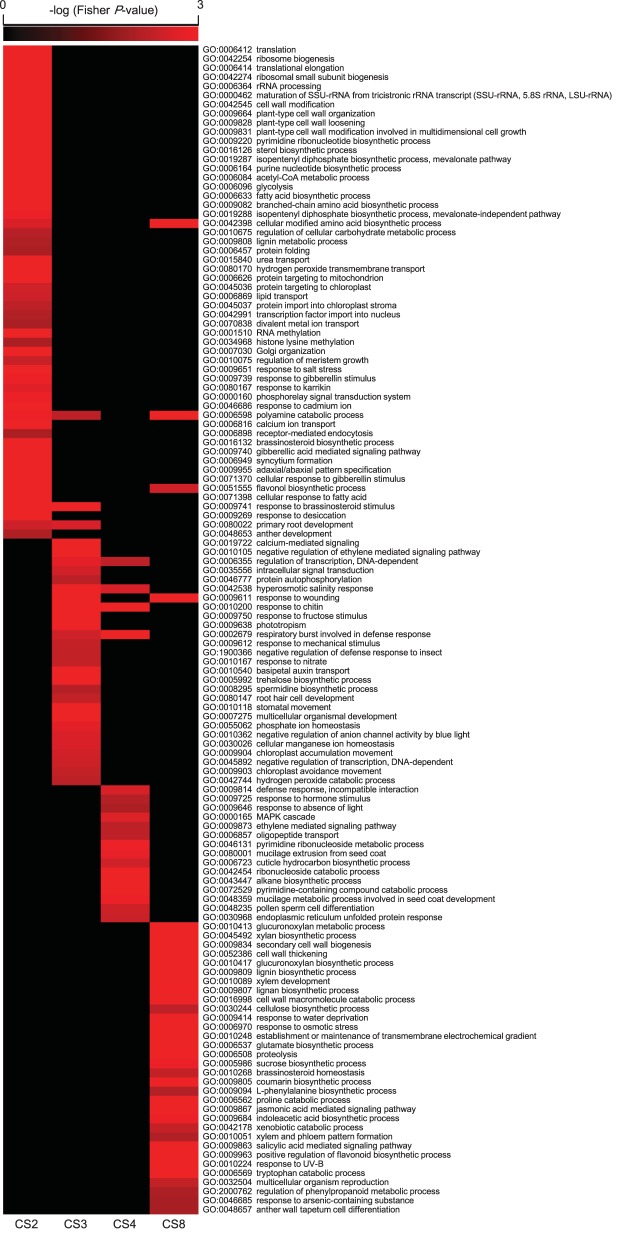
**Gene Ontology (GO) enrichment amongst *Alternanthera philoxeroides* differentially expressed genes in contrasting hydrological habitats**. This plot shows enriched GO terms (Fisher *P* < 0.01) in co-regulated groups: cluster 2 (CS2), CS3, CS4, and CS8 shown in the Figure 3. Supplementary Figure 5 shows enriched GO terms in all 11 clusters. Only the biological processes are showed.

Self-Organizing Maps (SOM) were used to identify co-regulated genes among the 7805 differentially expressed genes (Figure 3A). Eleven clusters of genes that share similar expression patterns were identified (Figure 3B). Figure 3C shows the temporal patterns of expression of 11 clusters. GO enrichment was performed to determine whether the co-regulated genes in each cluster were significantly associated with a specific biological process, cellular component or molecular function (Figure 4, Supplementary Figure 5, and Supplementary Table 6).

Cluster 2 (CS2) contained genes exhibiting sustained induction under the pond treatment. Their up-regulation began within 3 or 6 h and persisted throughout the 288 h experimental period. This cluster was enriched in genes involved in translation (Fisher *P*-value 1.00*E*-30), ribosome biogenesis (1.00*E*-30), RNA methylation (1.00*E*-30), and various organic substance metabolic processes, such as the pyrimidine ribonucleotide biosynthetic process (6.10*E*-13), the sterol biosynthetic process (2.40*E*-12), the isopentenyl diphosphate biosynthetic process (1.40*E*-06), and the purine nucleotide biosynthetic process (1.30*E*-05). CS2 was also enriched in genes associated with response to gibberellin stimulus (0.001) and cell wall modification (1.30*E*-10), including genes essential for cell wall loosening, such as *expansin* genes. In addition, CS2 had an overrepresentation of transport associated genes, including genes involved in urea transport (1.10*E*-08), hydrogen peroxide transmembrane transport (8.00*E*-05), and water transport (0.023). Notably, genes encoding *flavin-dependent monooxygenases* (*FMO*), whose transcripts accumulation has been identified as a marker for programmed cell death (Olszak et al., 2006), were also sustainedly up-regulated under the pond condition.

The genes in CS3 and CS4 exhibited strong induction beginning 1 h after the start of pond treatment, but their level of induction then decreased steadily and returned to the original levels. The genes in CS4 showed a faster decline in expression levels than those in CS3. Both CS3 and CS4 were enriched in genes associated phytohormone signaling pathways, especially for ethylene mediated signaling, such as group VII *ETHYLENE RESPONSE FACTOR* (*ERF*; homolog of *Arabidopsis RAP2.2*), *ETHYLENE RESPONSE* (*ETR*), and *ETHYLENE INSENSITIVE* (*EIN*). CS4 included a gene encoding the gibberellin receptor, GIBBERELLIN INSENSITIVE DWARF (GID). CS3 also included genes encode proteins related to calcium-mediated signaling (3.40*E*-06). Transcripts annotated as transcription factor and kinase/phosphatase genes were remarkably overrepresented in both clusters. About 8.14 and 11.69% of the co-regulated mRNAs in CS3 encoded transcription factors and kinases/phosphatase, respectively. The corresponding ratios in CS4 were 10.96 and 9.59%, respectively.

The genes in CS6 and CS7 were mostly down-regulated in the pond treatment. The genes in CS7 were transiently up-regulated at the start of the pond treatment but then exhibited sustained down-regulation. This cluster was enriched in genes associated with response to xenobiotic stimulus (6.40*E*-04), induced systemic resistance—jasmonic acid mediated signaling pathway (8.20*E*-04), systemic acquired resistance—salicylic acid mediated signaling pathway (2.08*E*-03), defense response to fungus (4.73*E*-03), response to insect (7.93*E*-03), response to bacterium (8.48*E*-03), and vacuolar protein processing (9.35*E*-03).

The genes of CS8 exhibited a significant “up-down” pattern of expression. The expression levels of CS8 genes peaked at 3 h of the pond treatment and dropping quickly thereafter. This cluster was significantly enriched in genes associated with several processes involved in cell wall strengthening, including the glucuronoxylan metabolic process (1.00*E*-28), the xylan biosynthetic process (1.10*E*-28), the glucuronoxylan biosynthetic process (2.10*E*-20), the lignin biosynthetic process (7.80*E*-11), the lignin biosynthetic process (8.00*E*-07), the cellulose biosynthetic process (0.005), secondary cell wall biogenesis (9.10*E*-26), cell wall thickening (5.60*E*-21), and xylem development (8.50*E*-08). CS8 was also enriched in genes linked to osmotic stress responses and osmotic solute accumulation, including response to water deprivation (1.60*E*-05), response to osmotic stress (3.30*E*-04), establishment or maintenance of transmembrane electrochemical gradient (1.20*E*-04), polyamine catabolic process (2.50*E*-05), glutamate biosynthetic process (1.60*E*-04), cellular modified amino acid biosynthetic process (2.40*E*-04), proteolysis (4.50*E*-04), and sucrose biosynthetic process (0.001).

### Diverse gene categories showing transcriptional responses to environmental change in *A. philoxeroides*

#### Cell wall modification-related genes

Cell volume expansion during elongation growth requires the loosening of cell walls. Many enzymes are responsible for this process, including expansins (EXPs), xyloglucan endotransglucosylase/hydrolases (XTHs), cellulases, and pectinesterases (Fry et al., 1992; Catoire et al., 1998; Rose and Bennett, 1999; Darley et al., 2001; Rose et al., 2002; Kende et al., 2004; Lee et al., 2010). Many genes encoding cell wall-loosening enzymes exhibited sustained induction under the pond treatment (Supplementary Figure 6), such as all 12 differentially expressed genes encoding α-expansins (EXPAs), and 19 genes encoding XTHs, two genes encoding pectinesterases, and four genes encoding cellulases.

#### Aquaporin and ion pump genes

Enlarging cells absorb large amounts of water *via* aquaporins. We identified 22 differentially expressed genes encoding aquaporin (Supplementary Figure 7). Notably, 14 of the 18 differentially expressed genes encoding the plasma membrane intrinsic protein (PIP) and the tonoplast intrinsic protein (TIP) were significantly up-regulated under pond condition. Cell enlargement growth is typically accompanied by the transport of inorganic ions *via* ion pumps. All four differentially expressed genes encoding plasma membrane H^+^-ATPase were up-regulated under the pond treatment (Supplementary Figure 8). In addition, one gene encoding plastid membrane H^+^-ATPase, two genes encoding vacuolar H^+^-PPase, and three genes encoding plasma membrane Ca^2+^-ATPase were also identified as the up-regulated genes in the stem internodes of *A. philoxeroides* under pond conditions (Supplementary Figure 8).

#### Cell death-related genes

The expression of 68 genes implicated in cell death differed significantly between two water habitats (Supplementary Figure 9). Many of these genes were associated with reactive oxygen species (ROS) generation and metabolic pathway. Genes encoding proteins involved in H_2_O_2_ generation exhibited consistent or late up-regulation under pond conditions, such as the aldehyde dehydrogenase and the VERNALIZATION5/VIN3-like protein (Supplementary Figure 9). Three of the four differentially expressed genes encoding catalases (CATs), which catalyze the degradation of H_2_O_2_, were down-regulated under pond conditions (Supplementary Figure 9). Genes encoding FMOs, metacaspase and homologs of *Arabidopsis* MYB30 were also found significantly up-regulated following submergence (Supplementary Figure 9).

#### Protein kinase, protein phosphatase, and transcription factor

Three hundred and forty-seven genes encoding protein kinases and sixty-three genes encoding protein phosphatases showed different expression patterns in the pond and upland treatments (Supplementary Figure 10 and Supplementary Table 7). In addition, 332 genes encoding transcription factors were differentially expressed in the two habitats. These transcription factors spanned 28 major families (Supplementary Figure 11A). Of them, the group VII ERF transcription factors have been shown to be associated with the elongation growth and adaptive anaerobic responses in submerged plants (Nakano et al., 2006; Hattori et al., 2009). Many ERF genes were strongly up-regulated in *A. philoxeroides* under pond conditions, including the group VII *ERF*s Contig23336 and ACluster3868_Consensus1 (Supplementary Figures 11B,C and Supplementary Table 8).

#### Calcium and hormone mediated signaling-related genes

One hundred and eight genes associated with calcium-mediated signaling exhibited different mRNA accumulation patterns in the two studied water habitats (Supplementary Figure 12). Of which, 70 were up-regulated under pond conditions, including genes encoding calmodulins (CAMs), CaM-like proteins (CMLs), calcineurin B-like proteins (CBLs), calmodulin-binding proteins, calcium-binding domain-containing proteins, calcium/calmodulin-dependent protein kinases, CBL-interacting protein kinases, and cyclic nucleotide-gated channels (CNGCs). We also observed significant transcriptional regulation of genes involved in multiple phytohormone-mediated signaling pathways (Supplementary Figure 13). Genes associated with ethylene signaling pathways were among those most strongly induced by the pond treatment. Of the 53 differentially expressed genes implicated in ethylene signaling, 26 were induced within 1 h of the start of the pond treatment. Twenty-three of the forty differentially expressed genes implicated in gibberellin signaling exhibited a late and sustained induction beginning 3 or 6 h after the start of pond treatment. Of 98 differentially expressed genes associated with abscisic acid signaling, 72 were significantly down-regulated under pond conditions.

### Comparative analysis of plastic responses between *A. philoxeroides* and *A. pungens*

**Figure 5:**
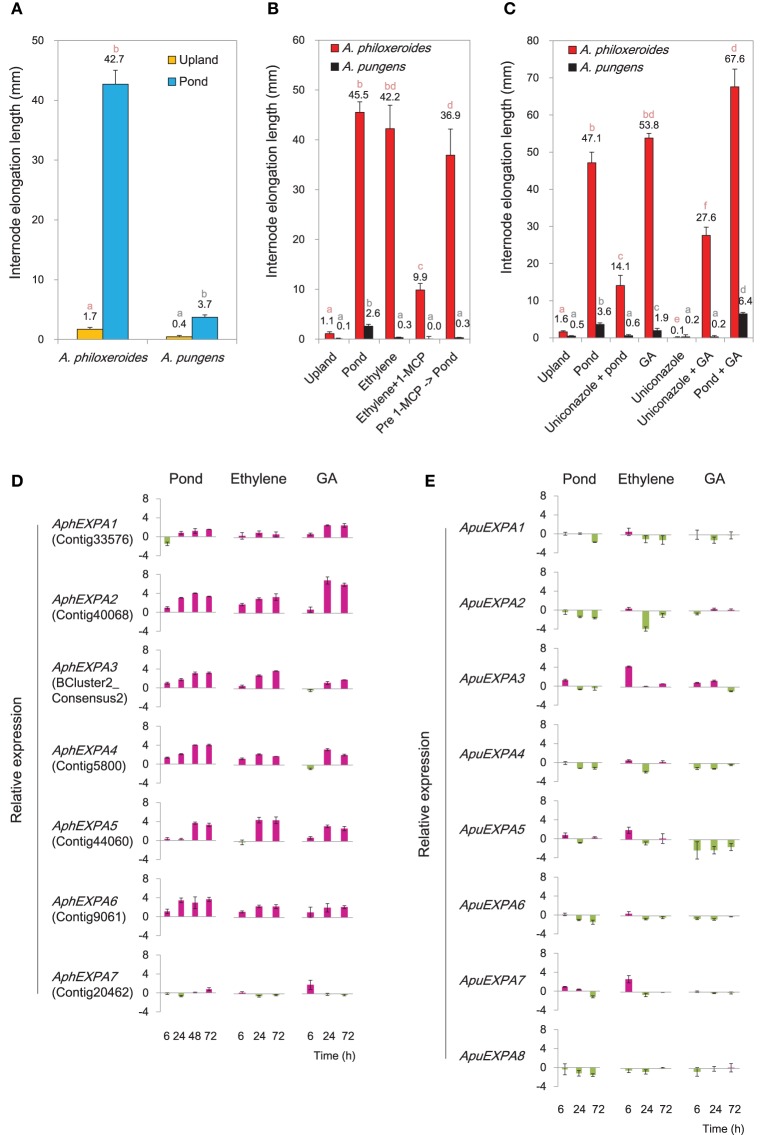
**Variations between *A. philoxeroides* and *A. pungens***. **(A)** Variation in internode elongation growth between two species. Quoted values are means + *s.d.*, *n* = 15. Plants were subjected to treatments for 15 days. **(B)** Varied responses to the ethylene treatment. Quoted values are means + *s.d.*, *n* = 15. **(C)** Varied responses to the GA treatment. Quoted values are means + *s.d.*, *n* = 15. Different lowercase letters on the columns in panels **(A–C)** indicate statistically significant differences among treatments in the same species, as judged by *t*-test (*P* < 0.05). **(D, E)** Accumulation patterns of α-expansin (EXPA) transcripts in *A. philoxeroides* **(D)** and *A. pungens* **(E)** under the pond, ethylene and GA treatments. Relative expression levels were calculated as log_2_(treatment/upland control). Quoted values are means + *s.d.*, *n* = 3. The corresponding unigene name in the reference transcriptome library of *A. philoxeroides* is given in parentheses.

Transcriptional profiling showed that genes involved in biosynthesis and signaling of ethylene and GA were highly induced under the pond treatment (Supplementary Figure 13). To verify their roles in mediating ecologically-relevant phenotypic responses, treatments with exogenous ethylene and GA and their inhibitors were carried out in common garden experiments. The results showed that both ethylene and GA induced appreciable internode elongation in *A. philoxeroides*, similar to that induced by submergence (Figures 5A–C). Meanwhile, the ethylene perception inhibitor 1-MCP and the GA biosynthesis inhibitor uniconazole both strongly suppressed internode elongation under pond conditions (Figures 5B,C), suggesting the appreciable internode elongation under pond conditions in *A. philoxeroides* required ethylene and GA perception. In contrast, the terricolous *A. pungens* exhibited a much less degree of internode elongation than *A. philoxeroides* under the pond treatment (Figure 5A). Likewise, the promotion by application of ethylene and GA was limited to internode elongation in *A. pungens* (Figures 5B,C).

Seven α-expansin genes were isolated from *A. philoxeroides* (*AphEXPA*s), and eight from *A. pungens* (*ApuEXPA*s; Supplementary Figure 14). The transcription levels of *A. philoxeroides EXPA*s (*AphEXPA1* to *AphEXPA6*) increased markedly under pond conditions except *AphEXPA7* (Figure 5D). Conversely, four of the eight *A. pungens EXPA*s (*ApuEXPA1, ApuEXPA2, ApuEXPA4, ApuEXPA8*) did not show any induction following submergence; three (*ApuEXPA3, ApuEXPA6, ApuEXPA7*) exhibited slight but transient up-regulation and were then down-regulated once more; and one (*ApuEXPA5*) exhibited a weakly fluctuating expression pattern (Figure 5E). Both ethylene and GA treatments caused sustained increases in transcript abundance of six *EXPA*s (*AphEXPA1* to *AphEXPA6*) in *A. philoxeroides* (Figure 5D). However, none of the *EXPA*s of *A. pungens* exhibited sustained induction in response to either ethylene or GA treatment (Figure 5E). Overall, the patterns of *EXPA* expression induced by ethylene and GA treatment were very similar to those observed following submergence, and correlated with the degree of elongation growth of internodes (Figures 5B–E).

**Figure 6:**
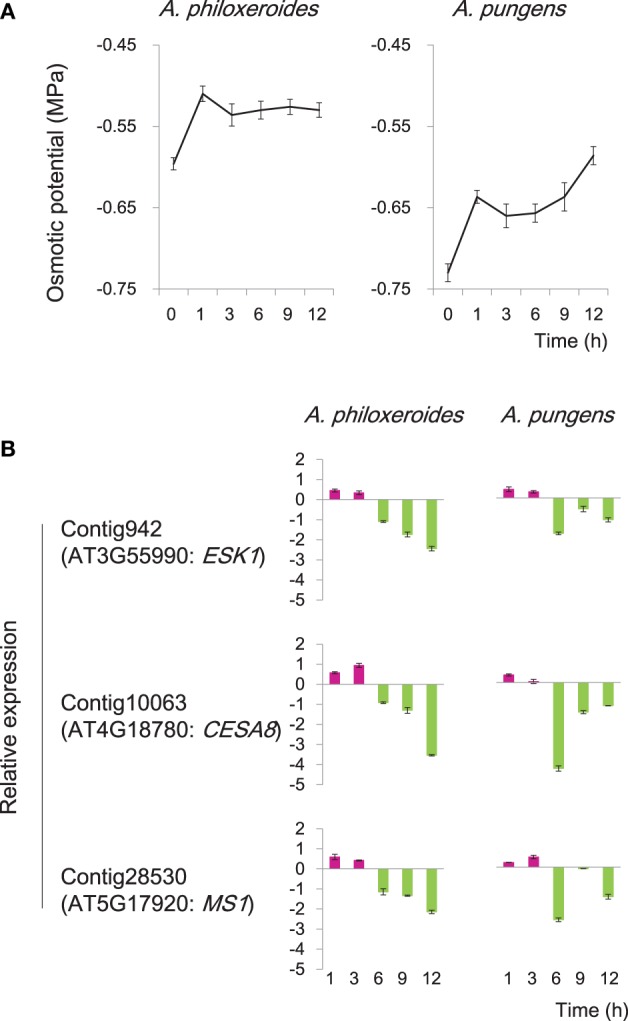
**Variation in cellular osmotic potential following submergence in *A. philoxeroides* and *A. pungens***. **(A)** Trends in the cell sap osmotic potential during the pond treatment in both species. Quoted values are means + *s.d.*, *n* = 8. **(B)** Expression patterns of genes involved in osmotic stress responses during the pond treatment. Relative expression levels were calculated as log_2_(pond/upland). Quoted values are means + *s.d.*, *n* = 3. The names of Arabidopsis homologous genes were shown in parentheses: *ESK1, ESKIMO 1*; *CESA8, cellulose synthase A8*; *MS1, methionine synthase 1*.

The induction of diverse osmostress response genes were detected at the early stage of the pond treatment (CS8; Figure 4). During the first 3 h of the pond treatment, the osmotic potential of the cell sap varied in an “up-down” fashion in both species (Figure 6A). Three genes homologous to *Arabidopsis ESK1, CESA8*, and *MS1*, respectively, which were predicted to be involved in osmotic stress response, were cloned and experimentally verified by qRT-PCR. The results showed that in both species, these genes exhibited an “up-down” expression pattern: the levels of their mRNAs increased during the first 3 h of the pond treatment and then fell rapidly thereafter (Figure 6B). This pattern was consistent with the variation of the cellular osmotic potential during the early hours of the pond treatment in both *A. philoxeroides* and *A. pungens* (Figure 6A). However, the two species subsequently exhibited very different trends in osmotic potential even though the expression patterns of related genes remained very similar. *A. philoxeroides* seemed able to exert control over the osmotic potential fluctuation and maintain a constant cellular osmotic potential during prolonged submergence. In contrast, the osmotic potential of *A. pungens* exhibited a sustained increase (Figure 6A).

## Discussion

Time course gene expression profiling revealed the genome wide transcriptional responses of *A. philoxeroides* to altered hydrological conditions. Identification of clusters of genes whose expression levels simultaneously rise and fall throughout a time course provided insights into the molecular and cellular processes underlying the development of plastic traits in *A. philoxeroides*. Putatively co-regulated biological processes and candidate genes potentially responsible for the plasticity can be identified based on analyses of reaction norms of gene expression.

### “Just-in-time” strengthening followed by sustained loosening and elongation growth of cell wall

Around 500 genes were transiently up-regulated early in the pond treatment but returned quickly (CS8; Figure 3C). Within this cluster, genes linked to secondary wall development and lignin biosynthesis were significantly enriched (Figure 4). The strong coordinated regulation of these genes suggested a “Just-in-time” response of cell wall to submergence. Cell wall thickening and depositions of lignin and suberin have been observed in the submerged roots of many species (Seago et al., 1999; Visser et al., 2000; Vasellati et al., 2001; Ryser et al., 2011). It has been reported that, upon flooding, plants can develop a barrier in roots by depositing new materials in cell walls to prevent radial loss of O_2_ to the soil (Colmer, 2003; Garthwaite et al., 2006; Shiono et al., 2011). However, such a barrier has never been reported in shoots. It is unclear whether the induced expression of the genes in CS8 is associated with the formation of the inducible barrier in the submerged shoots of *A. philoxeroides*.

Consistent with early up regulation of cell wall strengthening gene, we also observed increased expression of genes linked to synthesis of organic osmotic solutes in the first 3 h of the pond treatment (CS8; Figure 4). Moreover, the transient up-regulation of genes related to cell wall strengthening and osmotic solute accumulation coincided with the sharp increase and subsequent slight decrease of the cellular osmotic potential (Figure 6A), suggesting that these genes may jointly contribute to the observed adjustment and stabilization of osmotic potential. The nature of the relationship between cell wall strengthening and osmotic adjustment is unclear. It is notable, however, that the plant cell wall is a highly dynamic structure and serves many functions (Dzierzynska, 2006; Geitmann, 2006; Szymanski and Cosgrove, 2009). One of the critical functions is to prevent cell swelling because of osmotic pressure. The tensile strength of the cell wall allows plant cells to build up turgor pressure within cells, to equalize the osmotic pressure and prevent the further water influx. Normally, the cell wall is thickened and strengthened after cell elongation ceases. However, early activation of cell wall strengthening-related gene transcription can be elicited to prevent an unlimited increase in the cellular osmotic potential due to an oxygen deficit by increasing the pressure the cell wall exerts on the protoplast.

During the plastic development of *A. philoxeroides*, the cell wall system performs seemingly contradictory roles. On the one hand, it must be rigid enough to allow turgor pressure to build up, while on the other, it needs to be loosened in some way to permit cell enlargement during growth. Unlike the impulse patterns of cell wall-strengthening genes, genes encoding cell wall-loosening enzyme EXPAs, exhibited sustained up-regulation in *A*. *philoxeroides* following the pond treatment (CS2; Figure 4, Supplementary Figure 6). It has been suggested that expansins from the α-expansin subfamily are the most active cell wall loosening enzymes in dicots (Cosgrove, 2000; Lee et al., 2001). The enhanced expression of *EXPA*s under pond conditions agreed well with the increased growth of submerged internodes in *A*. *philoxeroides*.

### Active elongation growth accompanied by programmed cell death

*A. philoxeroides* exhibited a remarkable plasticity in internode elongation, which enables it to escape submergence and survive in pond habitats (Geng et al., 2007; Gao et al., 2010). Clustered gene expression profiles pointed to the joint contribution of groups of genes to the relevant cellular processes. Cell expansion during growth requires both cell wall loosening and rapid water influxes (Ooume et al., 2009). *EXPA*s involved in cell-wall loosening were strongly up-regulated under pond conditions (CS2; Figure 4, Supplementary Figure 6). PIP and TIP genes were also up-regulated following submergence (CS2; Supplementary Table 6 and Supplementary Figure 7). Previous studies have shown that PIPs are associated with elongation growth in higher plants by facilitating growth-associated water uptake into cells (O'Brien et al., 2002; Tyerman et al., 2002; Hachez et al., 2008; Muto et al., 2011). It has also been demonstrated that TIP expression directly promotes increases in vacuolar volume, which is tightly correlated with cell expansion (Ludevid et al., 1992; Chaumont et al., 1998; Reisen et al., 2003; Okubo-Kurihara et al., 2009). In addition, the accelerated water influx *via* PIPs and TIPs indirectly affects the accumulation of O_2_, CO_2_, micronutrients, and osmotic substances in the cell and thereby supplies the materials required for rapid plant growth (Tyerman et al., 2002; Reisen et al., 2003; Ma et al., 2004; Katsuhara and Hanba, 2008). Plasma membrane H^+^-ATPase genes were also up-regulated in the internodes of *A. philoxeroides* under pond conditions (Supplementary Figure 8), that might contribute to cell expansion during internode elongation in *A. philoxeroides* by generating a proton electrochemical gradient to provide a driving force for active transport of ions, metabolites and water (Fricke et al., 1997; Fricke and Flowers, 1998; Sze et al., 1999), or to facilitate the activity of EXPs and XTHs by providing the acidic pH (Frías et al., 1996; Shieh and Cosgrove, 1998).

In company with the induced high-level expression of genes related to elongation growth, genes associated with calcium-signaling and ROS metabolism, which involved in cell death, were also significantly differentially expressed between two habitats. Submergence induced the expression of H_2_O_2_ biosynthesis genes while reduced the expression of CAT genes (Supplementary Figure 9). The coordinated gene expression changes related to hydrogen peroxide metabolism can lead to greater H_2_O_2_ accumulation, which may subsequently activate cell death and lysis. Genes encoding FMOs, metacaspase and homologs of *Arabidopsis* MYB30 were also strongly induced following submergence (Supplementary Figure 9). Given that all of these genes are known to promote programmed cell death (Feys et al., 2001; Suarez et al., 2004; Olszak et al., 2006), their simultaneous up-regulation strongly implies that they all contributed to the formation of lysigenous aerenchyma during the extension of pith cavity in submerged *A. philoxeroides* shoots. The enlarged cavity may facilitate the efficient movement of gases (such as O_2_, CO_2_, and ethylene) within the plant, which is crucial for survival while submerged (Carr et al., 1995; Tao et al., 2009).

### Integration and coordination of enteral cues and endogenous signals in regulating plastic development

The pond treatment led to increased internode elongation in *A. philoxeroides*. Concomitantly, altered expressions of genes linked to hormone and calcium-mediated signaling were detected between treatments (Supplementary Figures 12, 13). Ethylene has been long recognized as an essential signaling molecule for triggering downstream signaling pathways in response to oxygen deprivation, especially for regulating aerenchyma formation and elongation growth during long-term adaptation (He et al., 1996; Drew et al., 2000; Hattori et al., 2009). In consistent with the postulated role of ethylene as an upstream trigger for target gene expression, many genes implicated in ethylene signaling were induced within 1 h under the pond condition with their expression gradually returning to basal levels (CS4; Figure 4, Supplementary Figure 13). Genes implicated in gibberellin signaling exhibited a late and sustained induction pattern under the pond treatment, which has been reported to be essential for elongation growth in response to anoxia (Rijnders et al., 1997; Hattori et al., 2009). The enhanced elongation observed in submerged plants could be reproduced by treatment with ethylene or GA (Figures 5B,C).

The co-regulated genes exhibiting strong induction at the early stage of the pond treatment were also significantly enriched in genes related to calcium-mediated signaling (CS3; Figure 4). Ca^2+^ is widely recognized as a second messenger in signal transduction pathways associated with response to anoxia (Tsuji et al., 2000; Baxter-Burrell et al., 2002), and proposed to be essential promoter of apoptosis in aerenchyma development (He et al., 1996; Drew et al., 2000; Rajhi et al., 2011).

To date, the actual signals generated by submergence and the mechanisms by which the environmental cues are sensed, integrated and transformed into a plastic response remain elusive. It has been proposed that ROS changes, pH changes, metabolic changes, and changes in the availability of nutrients could serve as signals (Dat et al., 2004; Stamm and Kumar, 2010). Decline in O_2_ level due to flooding is majorly responsible for triggering the plant response (Dat et al., 2004; Voesenek and Sasidharan, 2013). In fact, the first event that takes place while flooding is the increased presence of H_2_O, which would detrimentally affect the water flux, sap osmotic potential, and turgor pressure of submerged organs, as shown in this study (Figure 6A). It has been revealed that cells of bacteria and yeast use several cellular indicators of water availability (e.g., changes in turgor and changes in cell wall–plasma membrane connections) as potential signaling agents (Dat et al., 2004). A transmembrane hybrid-type histidine kinase has also been found in *Arabidopsis* to function as a putative osmosensor (Urao et al., 1999). Such osmosensors could rapidly perceive and transmit changes in cell water homeostasis and trigger an adaptive response. Therefore, although plants may not directly sense changes in surrounding H_2_O levels, they may perceive signals of flooding through changes in cellular water homeostasis. In this way, the physical stress (flooding) is converted into a physiological signal, triggering a signaling cascade which includes a network of hormones and other common secondary signaling molecules and eventually leading to morphological changes.

### Implications of varied plastic responses in *A. philoxeroides* and *A. pungens*

Plant survival and growth require the maintenance of an appropriate cellular osmotic potential. The similar expression patterns of three osmostress response genes and trends in the cellular osmotic potential observed at the early stage of the pond treatment in both *A. philoxeroides* and *A. pungens* (Figure 6) suggest that *Altemanthera* species share some common pathways for regulating cellular osmotic potential during the early “stress response” to submergence, which may be valuable for mitigating the impact of sudden unexpected changes in habitat. However, cellular osmotic adjustment during the early response to the pond treatment may NOT be a “prerequisite” for the internode growth plasticity.

Comparative analysis between *A. philoxeroides* and *A. pungens* showed that submergence and ethylene and GA treatments could induce significant internode elongation in *A. philoxeroides* but not in *A. pungens* (Figures 5A–C). In addition, the expression of *EXPA* genes exhibited a higher environmental-sensitive pattern in *A. philoxeroides* than in *A. pungens* (Figures 5D,E). These results indicate that the interspecific variation in plasticity between *A. philoxeroides* and *A. pungens* is not due to environmentally-mediated changes in hormone levels but to variations in the type and relative abundance of different signal transducers and receptors expressed in the target tissue. To approach hormone-mediated plasticity in an ecologically relevant context, it seems necessary to think beyond just environmental alteration of hormone production, and begin considering how environment conditions affect the network of components that comprise a hormone signaling pathway (Lema and Kitano, 2013).

## Conclusions

Plants of *A. philoxeroides* retained a high level of growth plasticity for adapting to diverse habitats with varying water availability. This adaptability was reliant on genome-wide transcriptional plasticity in response to environmental fluctuations. Previous conceptual and theoretical work predicted two kinds of genetic mechanisms responsible for phenotypic plasticity: (i) plasticity caused by shifts in the amount of transcripts from environmentally sensitive loci and (ii) plasticity caused by regulatory loci that exert environmentally dependent control over structural gene expression (Schlichting and Pigliucci, 1993, 1995; Via et al., 1995; Nijhout, 1999). The phenotypically plastic changes in *A. philoxeroides* seem to be under the control of both types of genetic mechanisms, with multiple transcription factor genes and genes related to ethylene and calcium signaling being induced at the early stage of the pond treatment while plenty of structural genes being up-regulated later or sustainably up-regulated during the entire period of treatment. Monitoring the change in expression patterns based on the clustering of genes with similar expression patterns throughout time provided the possibility for querying biological processes that range from various responses of cells to external signals and identifying the complete set of activated genes involved in a biological process. Gene expression plasticity may have alleviated the constraints by genetic impoverishment in *A. philoxeroides*, and promoted its distribution across multiple ecological contexts, enhancing its ecological breadth.

## Author contributions

LG designed the research, performed the wet lab work and the data analysis, interpreted bioinformatic results, and drafted the manuscript. YG participated in the design of the study and in the data analysis, and drafted the manuscript. HY participated in the data analysis. YH and JY conceived the idea, and participated in the design of the study and in interpreting bioinformatic results, and drafted the manuscript. All authors carefully read and approved the final manuscript.

## Funding

This project was supported by the National Basic Research Program (2009CB119201), National Natural Science Foundation of China (31070201, 31300189), and State Key Laboratory of Systematic and Evolutionary Botany (LSEB2011-08).

### Conflict of interest statement

The authors declare that the research was conducted in the absence of any commercial or financial relationships that could be construed as a potential conflict of interest.
