# Supplementary material for: Gene Expression Reaction Norms Unravel the Molecular and Cellular Processes Underpinning the Plastic Phenotypes of Alternanthera Philoxeroides in Contrasting Hydrological Conditions
Source: Front Plant Sci. 2015 Nov 12;6:991. doi: 10.3389/fpls.2015.00991 (PMC4641913; doi:10.3389/fpls.2015.00991)

Supplementary Figure 1. Tissue sections of *A. philoxeroides* stem internodes under upland and pond conditions. Stem internode sections were taken from plants which had been grown under the upland and pond conditions for 15 days. Left, Transverse sections; right, longitudinal sections. Asterisks indicate the stem pith cavity areas. Bar, 100 µm.

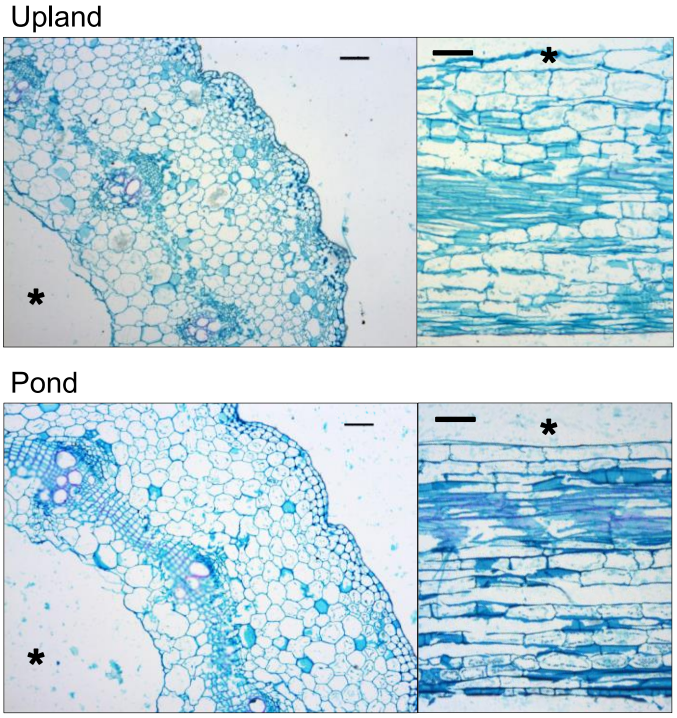

Supplement: Supplementary file 9 [file Image1.PDF]
