# Supplementary material for: Gene Expression Reaction Norms Unravel the Molecular and Cellular Processes Underpinning the Plastic Phenotypes of Alternanthera Philoxeroides in Contrasting Hydrological Conditions
Source: Front Plant Sci. 2015 Nov 12;6:991. doi: 10.3389/fpls.2015.00991 (PMC4641913; doi:10.3389/fpls.2015.00991)

Supplementary Figure 2. Cell death in *A. philoxeroides* stem pith cavities under upland and pond conditions. Stem pith cavity sections taken 48 h after the start of treatment were stained with Evans blue to detect cell death. Asterisks indicate the stem pith cavity areas. Bar, 1 mm.

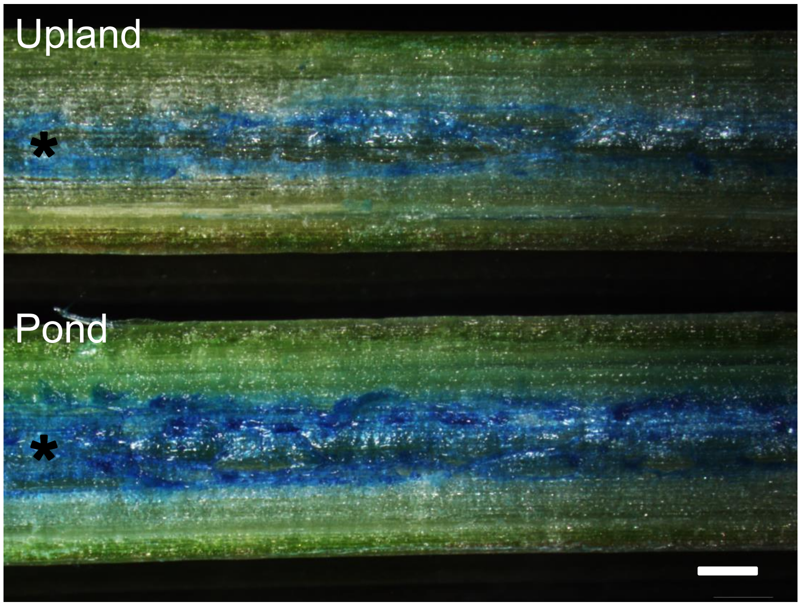

Supplement: Supplementary file 10 [file Image2.PDF]
