# Supplementary material for: Gene Expression Reaction Norms Unravel the Molecular and Cellular Processes Underpinning the Plastic Phenotypes of Alternanthera Philoxeroides in Contrasting Hydrological Conditions
Source: Front Plant Sci. 2015 Nov 12;6:991. doi: 10.3389/fpls.2015.00991 (PMC4641913; doi:10.3389/fpls.2015.00991)

Supplementary Figure 3. Gene Ontology (GO) classifications in *A. philoxeroides* (white) and *Arabidopsis* (black). **(A)** Biological process; **(B)** Cellular component; **(C)** Molecular function.

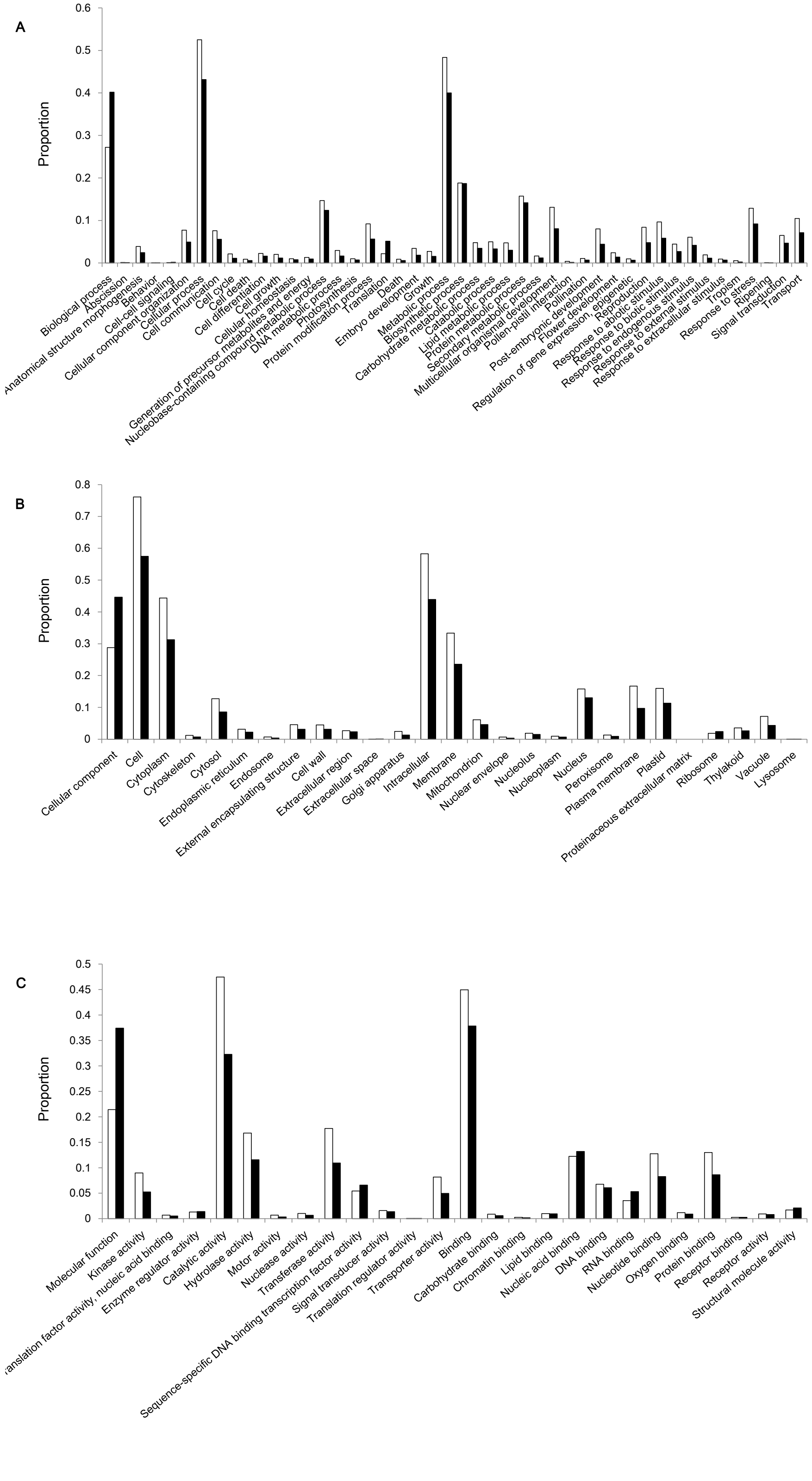

Supplement: Supplementary file 11 [file Image3.PDF]
