# Supplementary material for: Gene Expression Reaction Norms Unravel the Molecular and Cellular Processes Underpinning the Plastic Phenotypes of Alternanthera Philoxeroides in Contrasting Hydrological Conditions
Source: Front Plant Sci. 2015 Nov 12;6:991. doi: 10.3389/fpls.2015.00991 (PMC4641913; doi:10.3389/fpls.2015.00991)

Supplementary Figure 4. Confirmation of the expression profiles of 17 genes in *A. philoxeroides* by qRT-PCR. Gene expression data obtained by qRT-PCR and RNA sequencing are showed by blue bars and red bars, respectively. The *R* and *P* values indicate the correlation between the expression profiles determined by transcriptome sequencing and qRT-PCR. Relative expression levels were calculated as log_2(pond/upland) at the indicated time point.

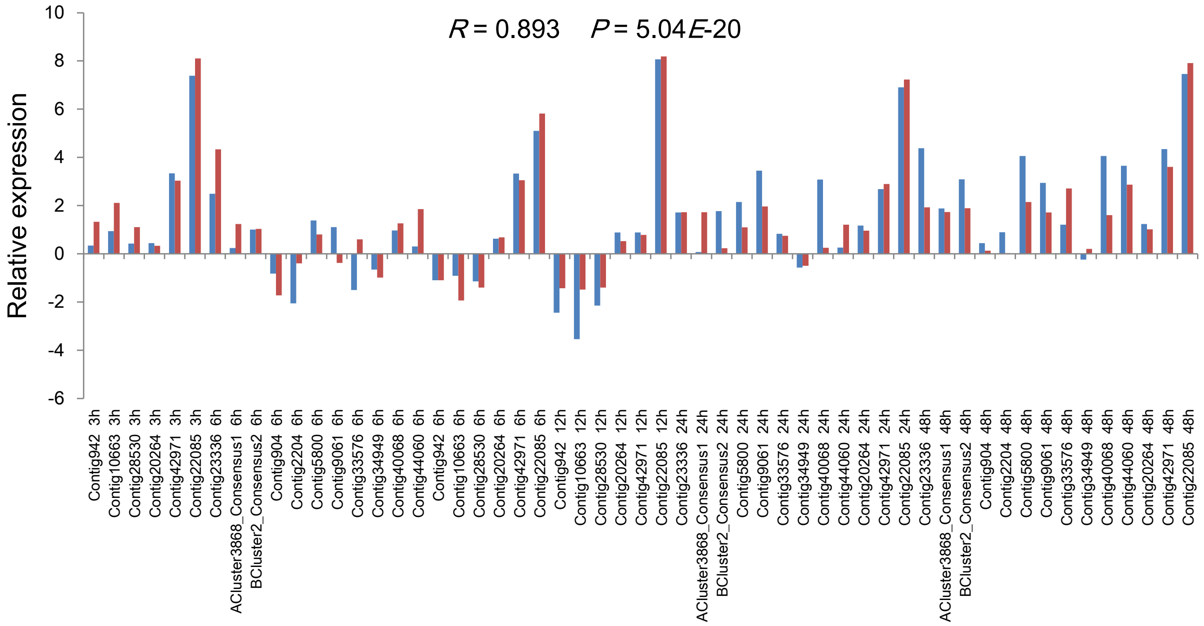

Supplement: Supplementary file 12 [file Image4.PDF]
