# Supplementary material for: Gene Expression Reaction Norms Unravel the Molecular and Cellular Processes Underpinning the Plastic Phenotypes of Alternanthera Philoxeroides in Contrasting Hydrological Conditions
Source: Front Plant Sci. 2015 Nov 12;6:991. doi: 10.3389/fpls.2015.00991 (PMC4641913; doi:10.3389/fpls.2015.00991)

Supplementary Figure 5. Gene Ontology (GO) enrichment amongst *A. philoxeroides* differentially expressed genes in contrasting hydrological habitats. This plot shows enriched GO terms (Fisher *P*-value < 0.01) in 11 co-regulated clusters shown in **Figure 3**. Only the biological processes are showed.

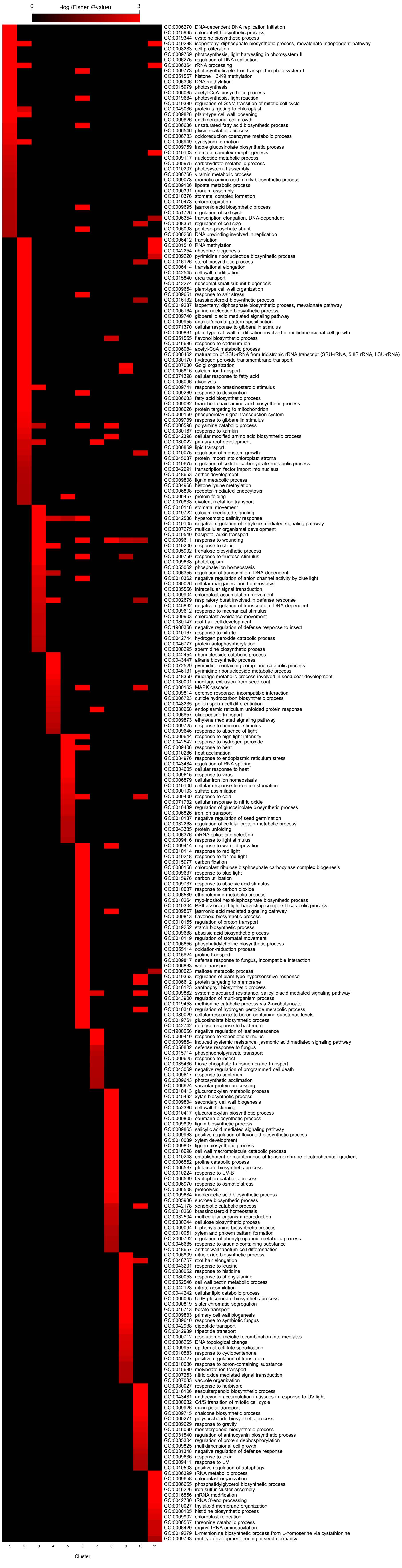

Supplement: Supplementary file 13 [file Image5.PDF]
