# Supplementary material for: Gene Expression Reaction Norms Unravel the Molecular and Cellular Processes Underpinning the Plastic Phenotypes of Alternanthera Philoxeroides in Contrasting Hydrological Conditions
Source: Front Plant Sci. 2015 Nov 12;6:991. doi: 10.3389/fpls.2015.00991 (PMC4641913; doi:10.3389/fpls.2015.00991)

**Supplementary Figure 7.** *A. philoxeroides* aquaporin genes that exhibited different expression patterns in contrasting hydrological habitats. Temporal patterns of expression changes during the time course of treatment were visualized using centroid linkage clustering method, with an uncentered correlation metric. $Log_2$(pond/upland) values were from **Supplementary Table 6**.

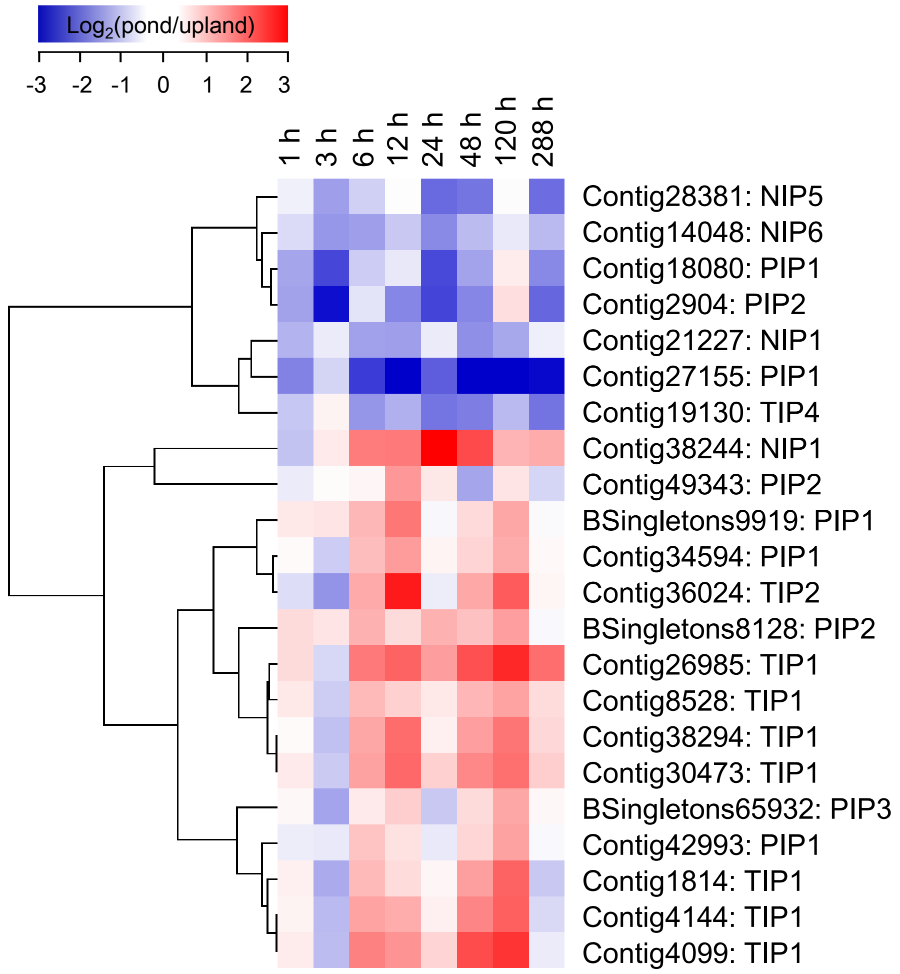

Supplement: Supplementary file 15 [file Image7.PDF]
