# Supplementary material for: Gene Expression Reaction Norms Unravel the Molecular and Cellular Processes Underpinning the Plastic Phenotypes of Alternanthera Philoxeroides in Contrasting Hydrological Conditions
Source: Front Plant Sci. 2015 Nov 12;6:991. doi: 10.3389/fpls.2015.00991 (PMC4641913; doi:10.3389/fpls.2015.00991)
Supplement: Supplementary file 18 [file Image10.PDF]

Supplementary Figure 10. *A. philoxeroides* protein kinase **(A)** and phosphatase **(B)** genes that exhibited different expression patterns in different water habitats. Temporal patterns of expression changes during the time course of treatment were visualized using centroid linkage clustering method, with an uncentered correlation metric. A list of protein kinase genes and annotations in **(A)** is provided in **Supplementary Table 7**. Log_2(pond/upland) values were from **Supplementary Table 6**.

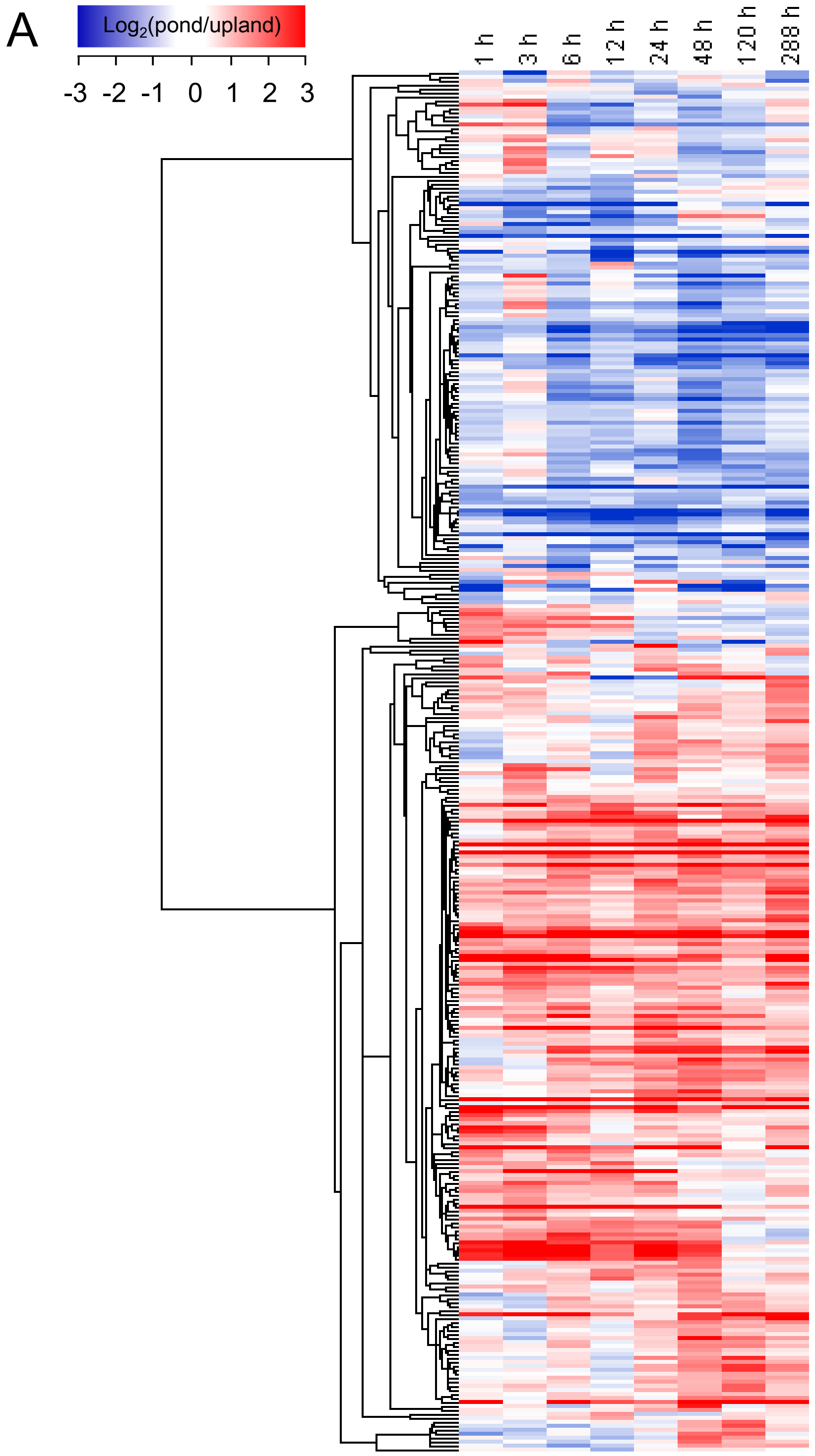

B

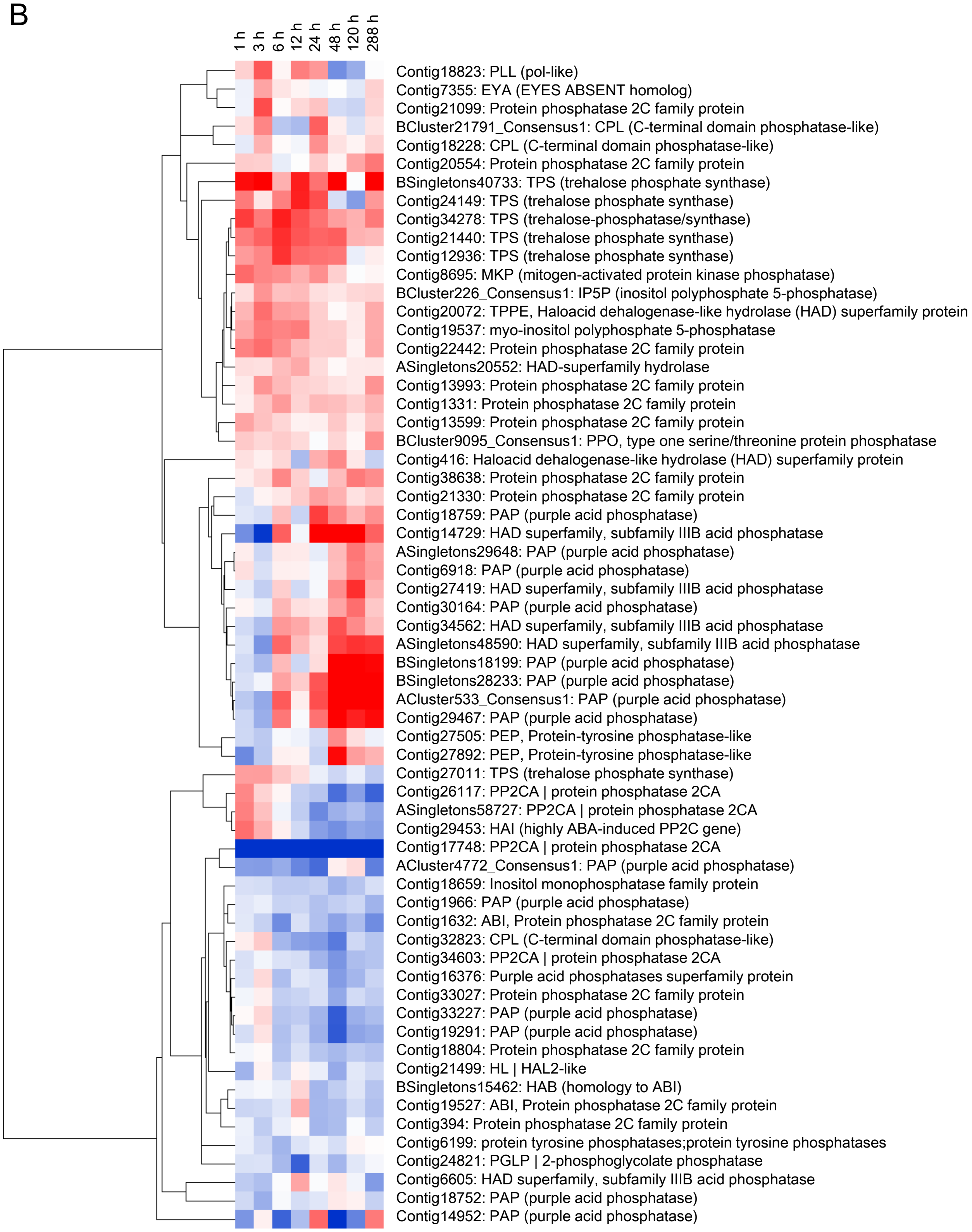

1 h
3 h
6 h
12 h
24 h
48 h
120 h
288 h
Contig18823: PLL (pol-like)
Contig7355: EYA (EYES ABSENT homolog)
Contig21099: Protein phosphatase 2C family protein
BCluster21791_Consensus1: CPL (C-terminal domain phosphatase-like)
Contig18228: CPL (C-terminal domain phosphatase-like)
Contig20554: Protein phosphatase 2C family protein
BSingletons40733: TPS (trehalose phosphate synthase)
Contig24149: TPS (trehalose phosphate synthase)
Contig34278: TPS (trehalose-phosphatase/synthase)
Contig21440: TPS (trehalose phosphate synthase)
Contig12936: TPS (trehalose phosphate synthase)
Contig8695: MKP (mitogen-activated protein kinase phosphatase)
BCluster226_Consensus1: IP5P (inositol polyphosphate 5-phosphatase)
Contig20072: TPPE, Haloacid dehalogenase-like hydrolase (HAD) superfamily protein
Contig19537: myo-inositol polyphosphate 5-phosphatase
Contig22442: Protein phosphatase 2C family protein
ASingletons20552: HAD-superfamily hydrolase
Contig13993: Protein phosphatase 2C family protein
Contig1331: Protein phosphatase 2C family protein
Contig13599: Protein phosphatase 2C family protein
BCluster9095_Consensus1: PPO, type one serine/threonine protein phosphatase
Contig416: Haloacid dehalogenase-like hydrolase (HAD) superfamily protein
Contig38638: Protein phosphatase 2C family protein
Contig21330: Protein phosphatase 2C family protein
Contig18759: PAP (purple acid phosphatase)
Contig14729: HAD superfamily, subfamily IIIB acid phosphatase
ASingletons29648: PAP (purple acid phosphatase)
Contig6918: PAP (purple acid phosphatase)
Contig27419: HAD superfamily, subfamily IIIB acid phosphatase
Contig30164: PAP (purple acid phosphatase)
Contig34562: HAD superfamily, subfamily IIIB acid phosphatase
ASingletons48590: HAD superfamily, subfamily IIIB acid phosphatase
BSingletons18199: PAP (purple acid phosphatase)
BSingletons28233: PAP (purple acid phosphatase)
ACluster533_Consensus1: PAP (purple acid phosphatase)
Contig29467: PAP (purple acid phosphatase)
Contig27505: PEP, Protein-tyrosine phosphatase-like
Contig27892: PEP, Protein-tyrosine phosphatase-like
Contig27011: TPS (trehalose phosphate synthase)
Contig26117: PP2CA | protein phosphatase 2CA
ASingletons58727: PP2CA | protein phosphatase 2CA
Contig29453: HAI (highly ABA-induced PP2C gene)
Contig17748: PP2CA | protein phosphatase 2CA
ACluster4772_Consensus1: PAP (purple acid phosphatase)
Contig18659: Inositol monophosphatase family protein
Contig1966: PAP (purple acid phosphatase)
Contig1632: ABI, Protein phosphatase 2C family protein
Contig32823: CPL (C-terminal domain phosphatase-like)
Contig34603: PP2CA | protein phosphatase 2CA
Contig16376: Purple acid phosphatases superfamily protein
Contig33027: Protein phosphatase 2C family protein
Contig33227: PAP (purple acid phosphatase)
Contig19291: PAP (purple acid phosphatase)
Contig18804: Protein phosphatase 2C family protein
Contig21499: HL | HAL2-like
BSingletons15462: HAB (homology to ABI)
Contig19527: ABI, Protein phosphatase 2C family protein
Contig394: Protein phosphatase 2C family protein
Contig6199: protein tyrosine phosphatases;protein tyrosine phosphatases
Contig24821: PGLP | 2-phosphoglycolate phosphatase
Contig6605: HAD superfamily, subfamily IIIB acid phosphatase
Contig18752: PAP (purple acid phosphatase)
Contig14952: PAP (purple acid phosphatase)
