# Supplementary material for: Gene Expression Reaction Norms Unravel the Molecular and Cellular Processes Underpinning the Plastic Phenotypes of Alternanthera Philoxeroides in Contrasting Hydrological Conditions
Source: Front Plant Sci. 2015 Nov 12;6:991. doi: 10.3389/fpls.2015.00991 (PMC4641913; doi:10.3389/fpls.2015.00991)
Supplement: Supplementary file 19 [file Image11.PDF]

Supplementary Figure 11. *A. philoxeroides* transcription factor family members that exhibited different expression patterns in different water habitats. **(A)** Number of differentially expressed TF family members. **(B)** Temporal patterns of expression changes of TF transcripts during the time course of treatment were visualized using centroid linkage clustering method, with an uncentered correlation metric. Log_2(pond/upland) values were from **Supplementary Table 6**. **(C)** Phylogenetic tree of *A. philoxeroides* ERF proteins based on a comparison of the AP2/ERF domain to representative *Arabidopsis* ERF genes. The amino acid sequences were aligned by ClustalW, and the phylogenetic tree was constructed using the NJ method. ERF of *A. philoxeroides* are labeled by solid circles. A list of *Arabidopsis* genes used for tree construction is provided in **Supplementary Table 8**. The names of the *Arabidopsis* ERFs are indicated based on the previous report (Nakano *et al*. 2006). Nakano T, Suzuki K, Fujimura T, Shinshi H. 2006. Genome-wide analysis of the ERF gene family in Arabidopsis and rice. *Plant Physiology* 140: 411-432.

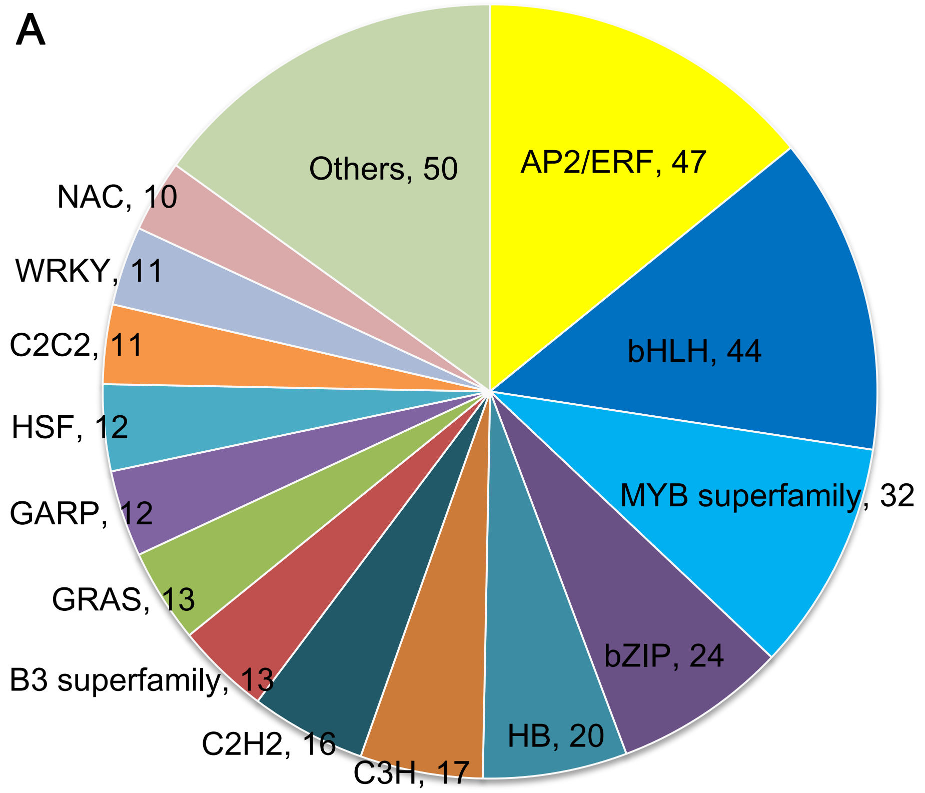

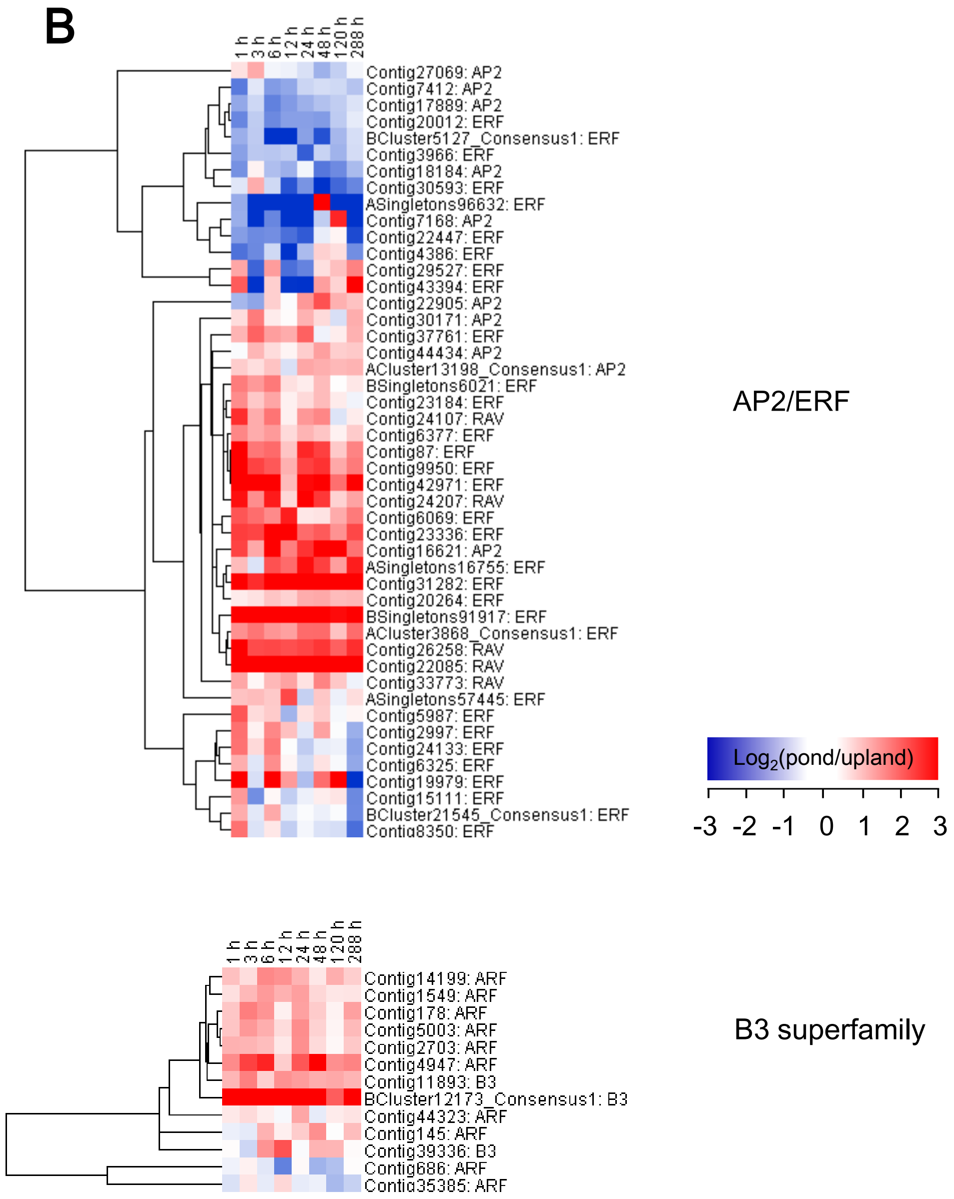
B
1 h
3 h
6 h
12 h
24 h
48 h
120 h
288 h
Contig27069: AP2
Contig7412: AP2
Contig17889: AP2
Contig20012: ERF
BCluster5127_Consensus1: ERF
Contig3966: ERF
Contig18184: AP2
Contig30593: ERF
ASingletons96632: ERF
Contig7168: AP2
Contig22447: ERF
Contig4386: ERF
Contig29527: ERF
Contig43394: ERF
Contig22905: AP2
Contig30171: AP2
Contig37761: ERF
Contig44434: AP2
ACluster13198_Consensus1: AP2
BSingletons6021: ERF
Contig23184: ERF
Contig24107: RAV
Contig6377: ERF
Contig87: ERF
Contig9950: ERF
Contig42971: ERF
Contig24207: RAV
Contig6069: ERF
Contig23336: ERF
Contig16621: AP2
ASingletons16755: ERF
Contig31282: ERF
Contig20264: ERF
BSingletons91917: ERF
ACluster3868_Consensus1: ERF
Contig26258: RAV
Contig22085: RAV
Contig33773: RAV
ASingletons57445: ERF
Contig5987: ERF
Contig2997: ERF
Contig24133: ERF
Contig6325: ERF
Contig19979: ERF
Contig15111: ERF
BCluster21545_Consensus1: ERF
Contig8350: ERF
AP2/ERF
Log2(pond/upland)
-3
-2
-1
0
1
2
3
1 h
3 h
6 h
12 h
24 h
48 h
120 h
288 h
Contig14199: ARF
Contig1549: ARF
Contig178: ARF
Contig5003: ARF
Contig2703: ARF
Contig4947: ARF
Contig11893: B3
BCluster12173_Consensus1: B3
Contig44323: ARF
Contig145: ARF
Contig39336: B3
Contig686: ARF
Contig35385: ARF
B3 superfamily

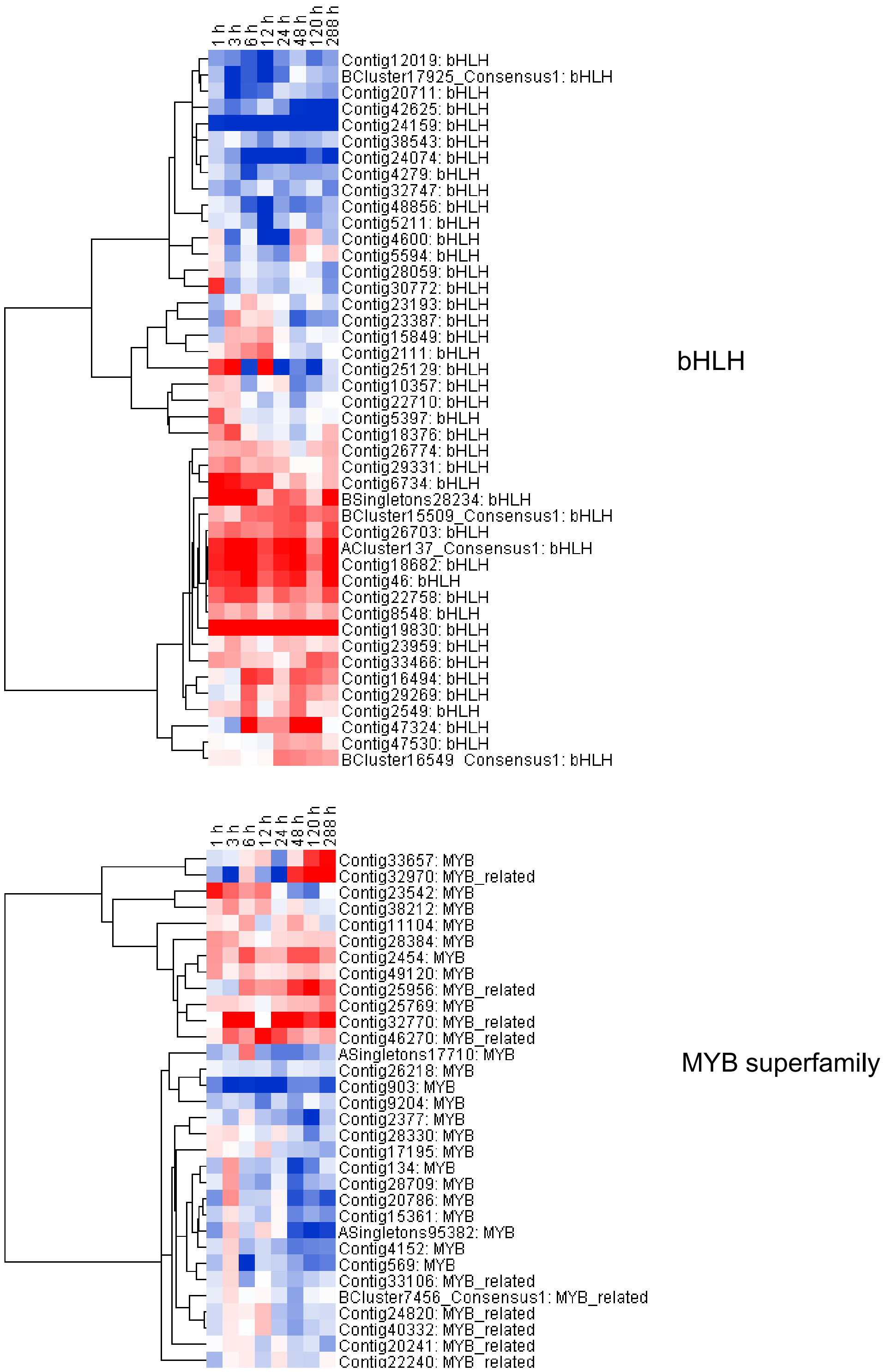
1 h
3 h
6 h
12 h
24 h
48 h
120 h
288 h
Contig12019: bHLH
BCluster17925_Consensus1: bHLH
Contig20711: bHLH
Contig42625: bHLH
Contig24159: bHLH
Contig38543: bHLH
Contig24074: bHLH
Contig4279: bHLH
Contig32747: bHLH
Contig48856: bHLH
Contig5211: bHLH
Contig4600: bHLH
Contig5594: bHLH
Contig28059: bHLH
Contig30772: bHLH
Contig23193: bHLH
Contig23387: bHLH
Contig15849: bHLH
Contig2111: bHLH
Contig25129: bHLH
Contig10357: bHLH
Contig22710: bHLH
Contig5397: bHLH
Contig18376: bHLH
Contig26774: bHLH
Contig29331: bHLH
Contig6734: bHLH
BSingletons28234: bHLH
BCluster15509_Consensus1: bHLH
Contig26703: bHLH
ACluster137_Consensus1: bHLH
Contig18682: bHLH
Contig46: bHLH
Contig22758: bHLH
Contig8548: bHLH
Contig19830: bHLH
Contig23959: bHLH
Contig33466: bHLH
Contig16494: bHLH
Contig29269: bHLH
Contig2549: bHLH
Contig47324: bHLH
Contig47530: bHLH
BCluster16549_Consensus1: bHLH
bHLH
1 h
3 h
6 h
12 h
24 h
48 h
120 h
288 h
Contig33657: MYB
Contig32970: MYB_related
Contig23542: MYB
Contig38212: MYB
Contig11104: MYB
Contig28384: MYB
Contig2454: MYB
Contig49120: MYB
Contig25956: MYB_related
Contig25769: MYB
Contig32770: MYB_related
Contig46270: MYB_related
ASingletons17710: MYB
Contig26218: MYB
Contig903: MYB
Contig9204: MYB
Contig2377: MYB
Contig28330: MYB
Contig17195: MYB
Contig134: MYB
Contig28709: MYB
Contig20786: MYB
Contig15361: MYB
ASingletons95382: MYB
Contig4152: MYB
Contig569: MYB
Contig33106: MYB_related
BCluster7456_Consensus1: MYB_related
Contig24820: MYB_related
Contig40332: MYB_related
Contig20241: MYB_related
Contig22240: MYB_related
MYB superfamily

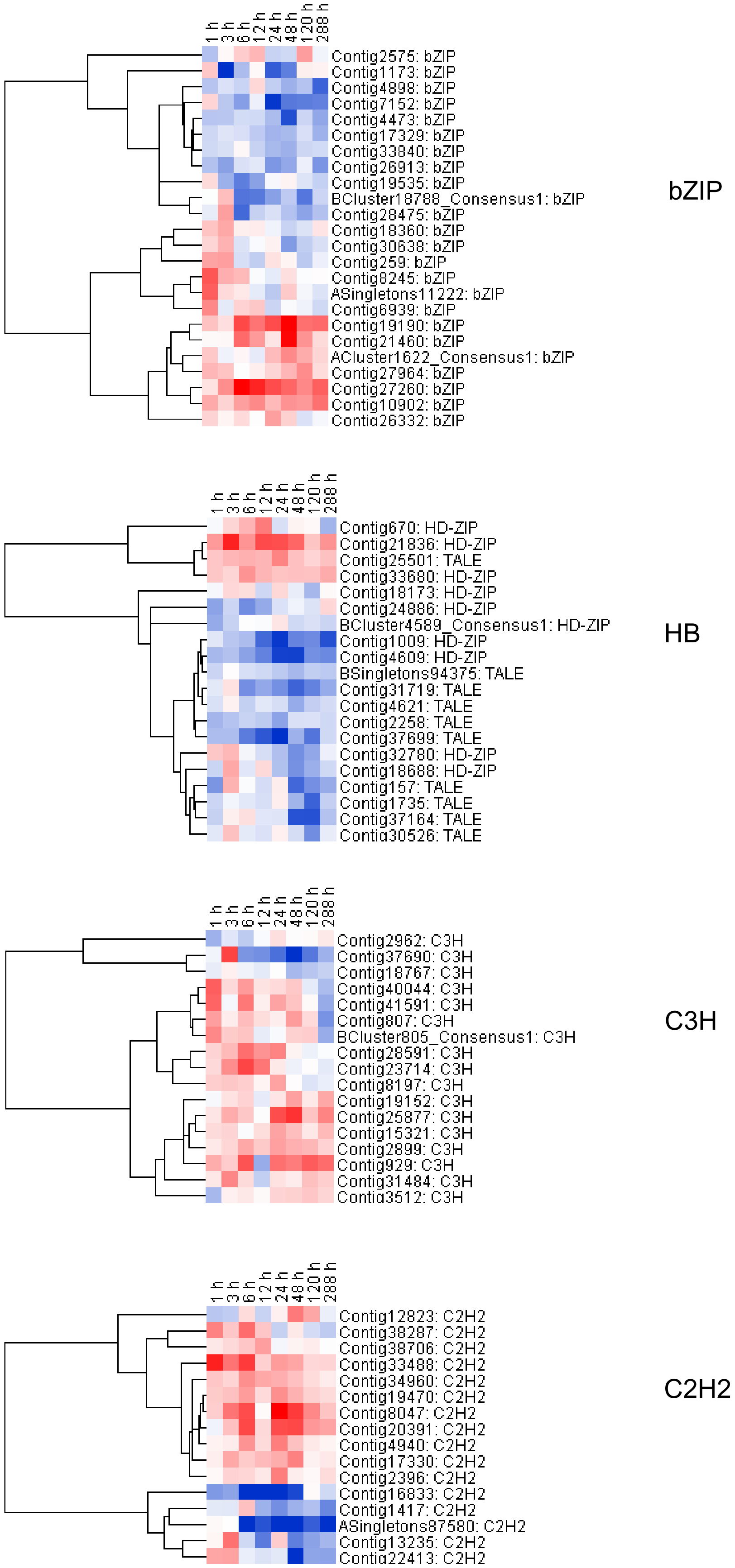

1 h
3 h
6 h
12 h
24 h
48 h
120 h
288 h
Contig2575: bZIP
Contig1173: bZIP
Contig4898: bZIP
Contig7152: bZIP
Contig4473: bZIP
Contig17329: bZIP
Contig33840: bZIP
Contig26913: bZIP
Contig19535: bZIP
BCluster18788_Consensus1: bZIP
Contig28475: bZIP
Contig18360: bZIP
Contig30638: bZIP
Contig259: bZIP
Contig8245: bZIP
ASingletons11222: bZIP
Contig6939: bZIP
Contig19190: bZIP
Contig21460: bZIP
ACluster1622_Consensus1: bZIP
Contig27964: bZIP
Contig27260: bZIP
Contig10902: bZIP
Contig26332: bZIP
bZIP
Contig670: HD-ZIP
Contig21836: HD-ZIP
Contig25501: TALE
Contig33680: HD-ZIP
Contig18173: HD-ZIP
Contig24886: HD-ZIP
BCluster4589_Consensus1: HD-ZIP
Contig1009: HD-ZIP
Contig4609: HD-ZIP
BSingletons94375: TALE
Contig31719: TALE
Contig4621: TALE
Contig2258: TALE
Contig37699: TALE
Contig32780: HD-ZIP
Contig18688: HD-ZIP
Contig157: TALE
Contig1735: TALE
Contig37164: TALE
Contig30526: TALE
HB
Contig2962: C3H
Contig37690: C3H
Contig18767: C3H
Contig40044: C3H
Contig41591: C3H
Contig807: C3H
BCluster805_Consensus1: C3H
Contig28591: C3H
Contig23714: C3H
Contig8197: C3H
Contig19152: C3H
Contig25877: C3H
Contig15321: C3H
Contig2899: C3H
Contig929: C3H
Contig31484: C3H
Contig3512: C3H
C3H
Contig12823: C2H2
Contig38287: C2H2
Contig38706: C2H2
Contig33488: C2H2
Contig34960: C2H2
Contig19470: C2H2
Contig8047: C2H2
Contig20391: C2H2
Contig4940: C2H2
Contig17330: C2H2
Contig2396: C2H2
Contig16833: C2H2
Contig1417: C2H2
ASingletons87580: C2H2
Contig13235: C2H2
Contig22413: C2H2
C2H2

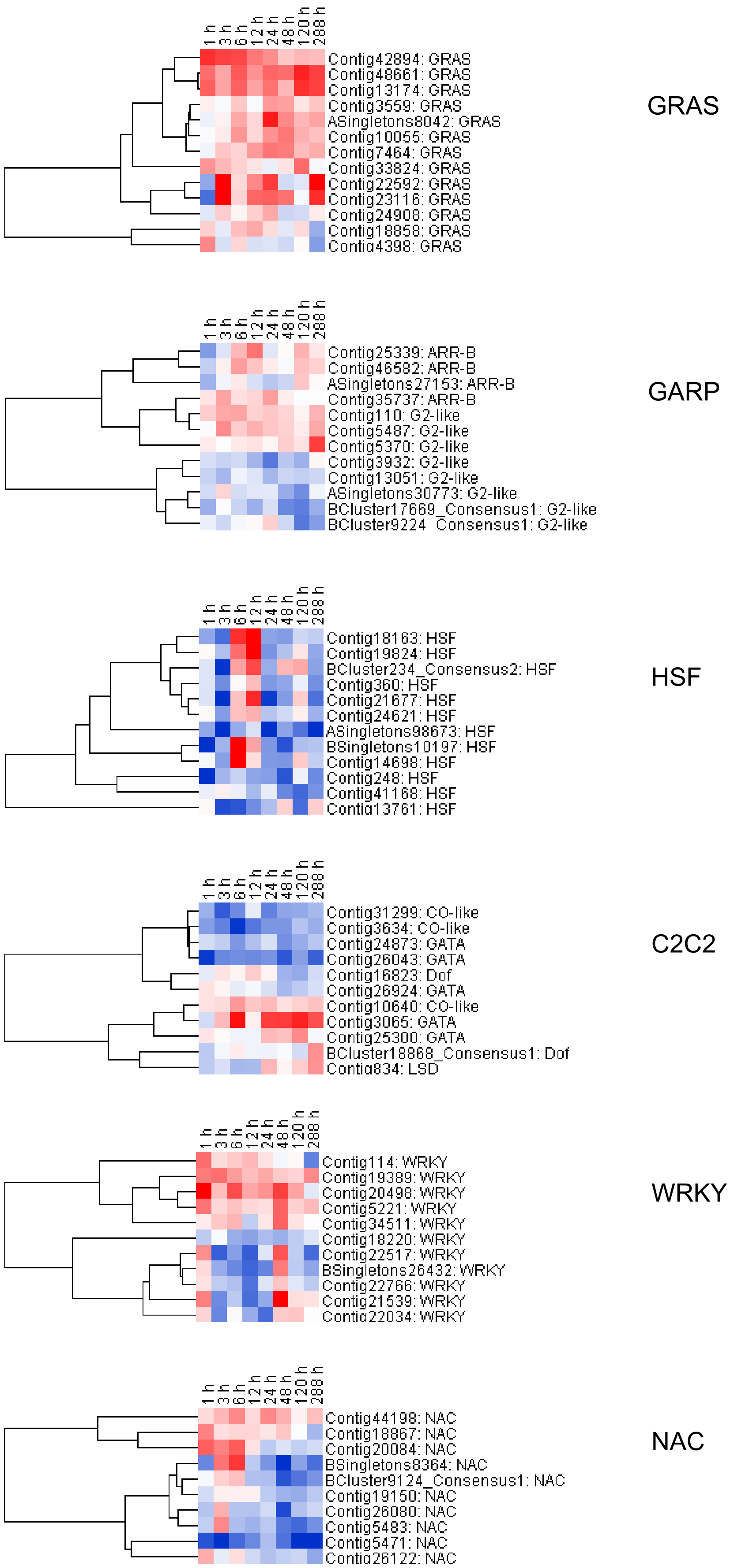
1 h
3 h
6 h
12 h
24 h
48 h
120 h
288 h
Contig42894: GRAS
Contig48661: GRAS
Contig13174: GRAS
Contig3559: GRAS
ASingletons8042: GRAS
Contig10055: GRAS
Contig7464: GRAS
Contig33824: GRAS
Contig22592: GRAS
Contig23116: GRAS
Contig24908: GRAS
Contig18858: GRAS
Contig4398: GRAS
GRAS
1 h
3 h
6 h
12 h
24 h
48 h
120 h
288 h
Contig25339: ARR-B
Contig46582: ARR-B
ASingletons27153: ARR-B
Contig35737: ARR-B
Contig110: G2-like
Contig5487: G2-like
Contig5370: G2-like
Contig3932: G2-like
Contig13051: G2-like
ASingletons30773: G2-like
BCluster17669_Consensus1: G2-like
BCluster9224_Consensus1: G2-like
GARP
1 h
3 h
6 h
12 h
24 h
48 h
120 h
288 h
Contig18163: HSF
Contig19824: HSF
BCluster234_Consensus2: HSF
Contig360: HSF
Contig21677: HSF
Contig24621: HSF
ASingletons98673: HSF
BSingletons10197: HSF
Contig14698: HSF
Contig248: HSF
Contig41168: HSF
Contig13761: HSF
HSF
1 h
3 h
6 h
12 h
24 h
48 h
120 h
288 h
Contig31299: CO-like
Contig3634: CO-like
Contig24873: GATA
Contig26043: GATA
Contig16823: Dof
Contig26924: GATA
Contig10640: CO-like
Contig3065: GATA
Contig25300: GATA
BCluster18868_Consensus1: Dof
Contig834: LSD
C2C2
1 h
3 h
6 h
12 h
24 h
48 h
120 h
288 h
Contig114: WRKY
Contig19389: WRKY
Contig20498: WRKY
Contig5221: WRKY
Contig34511: WRKY
Contig18220: WRKY
Contig22517: WRKY
BSingletons26432: WRKY
Contig22766: WRKY
Contig21539: WRKY
Contig22034: WRKY
WRKY
1 h
3 h
6 h
12 h
24 h
48 h
120 h
288 h
Contig44198: NAC
Contig18867: NAC
Contig20084: NAC
BSingletons8364: NAC
BCluster9124_Consensus1: NAC
Contig19150: NAC
Contig26080: NAC
Contig5483: NAC
Contig5471: NAC
Contig26122: NAC
NAC

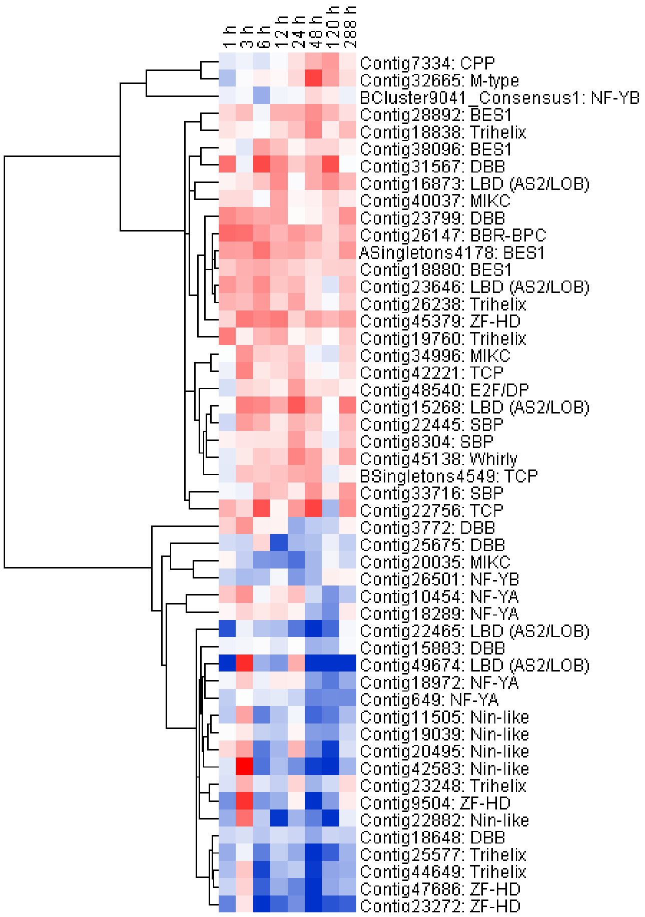
1 h
3 h
6 h
12 h
24 h
48 h
120 h
288 h
Contig7334: CPP
Contig32665: M-type
BCluster9041_Consensus1: NF-YB
Contig28892: BES1
Contig18838: Trihelix
Contig38096: BES1
Contig31567: DBB
Contig16873: LBD (AS2/LOB)
Contig40037: MIKC
Contig23799: DBB
Contig26147: BBR-BPC
ASingletons4178: BES1
Contig18880: BES1
Contig23646: LBD (AS2/LOB)
Contig26238: Trihelix
Contig45379: ZF-HD
Contig19760: Trihelix
Contig34996: MIKC
Contig42221: TCP
Contig48540: E2F/DP
Contig15268: LBD (AS2/LOB)
Contig22445: SBP
Contig8304: SBP
Contig45138: Whirly
BSingletons4549: TCP
Contig33716: SBP
Contig22756: TCP
Contig3772: DBB
Contig25675: DBB
Contig20035: MIKC
Contig26501: NF-YB
Contig10454: NF-YA
Contig18289: NF-YA
Contig22465: LBD (AS2/LOB)
Contig15883: DBB
Contig49674: LBD (AS2/LOB)
Contig18972: NF-YA
Contig649: NF-YA
Contig11505: Nin-like
Contig19039: Nin-like
Contig20495: Nin-like
Contig42583: Nin-like
Contig23248: Trihelix
Contig9504: ZF-HD
Contig22882: Nin-like
Contig18648: DBB
Contig25577: Trihelix
Contig44649: Trihelix
Contig47686: ZF-HD
Contig23272: ZF-HD

Others

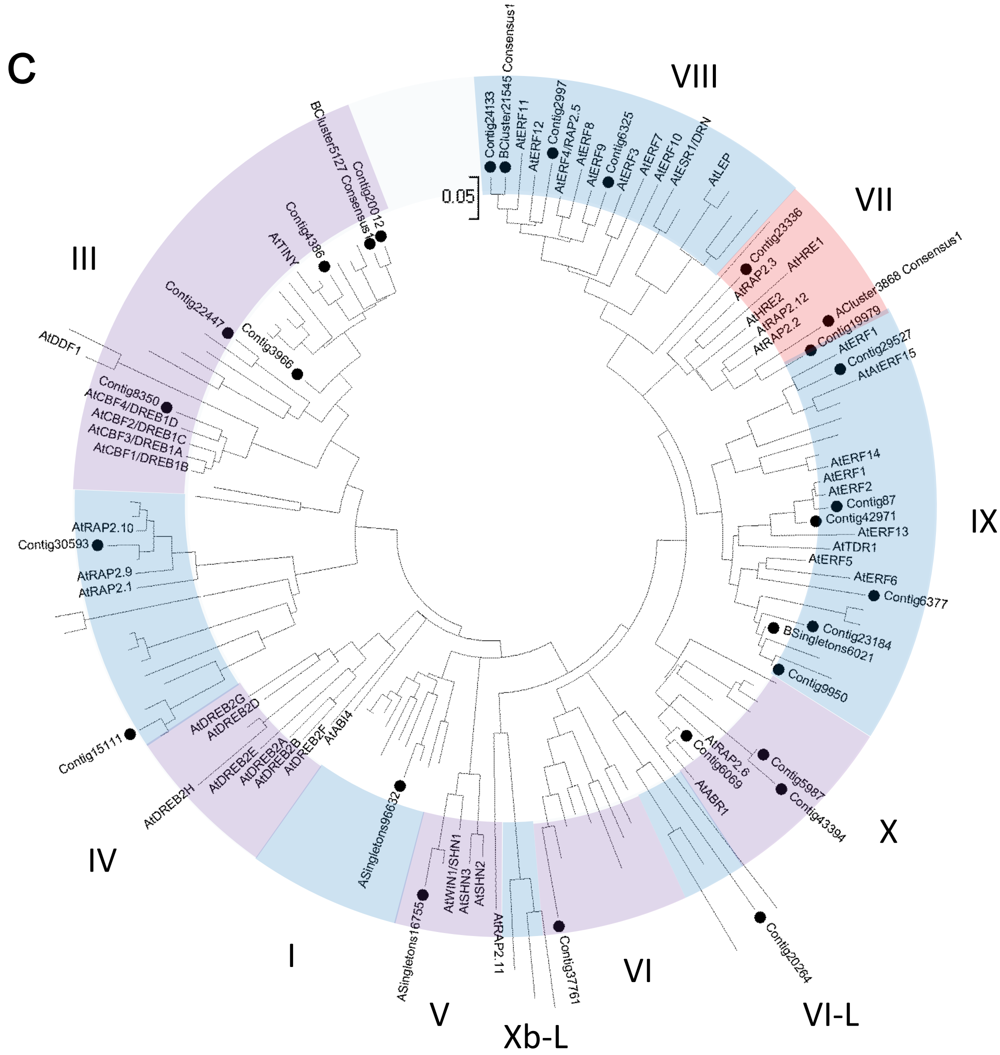
C
0.05
VIII
VII
IX
X
VI-L
VI
Xb-L
V
I
IV
II
III
Contig24133
BCluster21545 Consensus1
AtERF11
AtERF12
Contig2997
AtERF4/RAP2.5
AtERF8
AtERF9
Contig6325
AtERF3
AtERF7
AtERF10
AtESR1/DRN
AtLEP
Contig23336
AtRAP2.3
AtHRE1
AtHRE2
AtRAP2.12
AtRAP2.2
ACluster3868 Consensus1
Contig19979
AtERF1
Contig29527
AtAtERF15
AtERF14
AtERF1
AtERF2
Contig87
Contig42971
AtERF13
AtTDR1
AtERF5
AtERF6
Contig6377
BSingletons6021
Contig23184
Contig9950
Contig5987
Contig43394
AtRAP2.6
Contig6069
AtABR1
Contig20264
Contig37761
AtRAP2.11
AtWIN1/SHN1
AtSHN3
AtSHN2
ASingletons16755
ASingletons96632
AtABI4
AtDREB2F
AtDREB2B
AtDREB2A
AtDREB2E
AtDREB2H
AtDREB2G
AtDREB2D
Contig15111
AtRAP2.10
Contig30593
AtRAP2.9
AtRAP2.1
AtDDF1
Contig8350
AtCBF4/DREB1D
AtCBF2/DREB1C
AtCBF3/DREB1A
AtCBF1/DREB1B
Contig22447
Contig3966
AtTINY
Contig4386
BCluster5127 Consensus1
Contig20012
