# Supplementary material for: Gene Expression Reaction Norms Unravel the Molecular and Cellular Processes Underpinning the Plastic Phenotypes of Alternanthera Philoxeroides in Contrasting Hydrological Conditions
Source: Front Plant Sci. 2015 Nov 12;6:991. doi: 10.3389/fpls.2015.00991 (PMC4641913; doi:10.3389/fpls.2015.00991)

Supplementary Figure 14. Phylogenetic tree showing the similarities of the deduced amino acid sequences of *AphEXPA*s and *ApuEXPA*s. The amino acid sequences were aligned by ClustalW, and the phylogenetic tree was constructed using the NJ method.

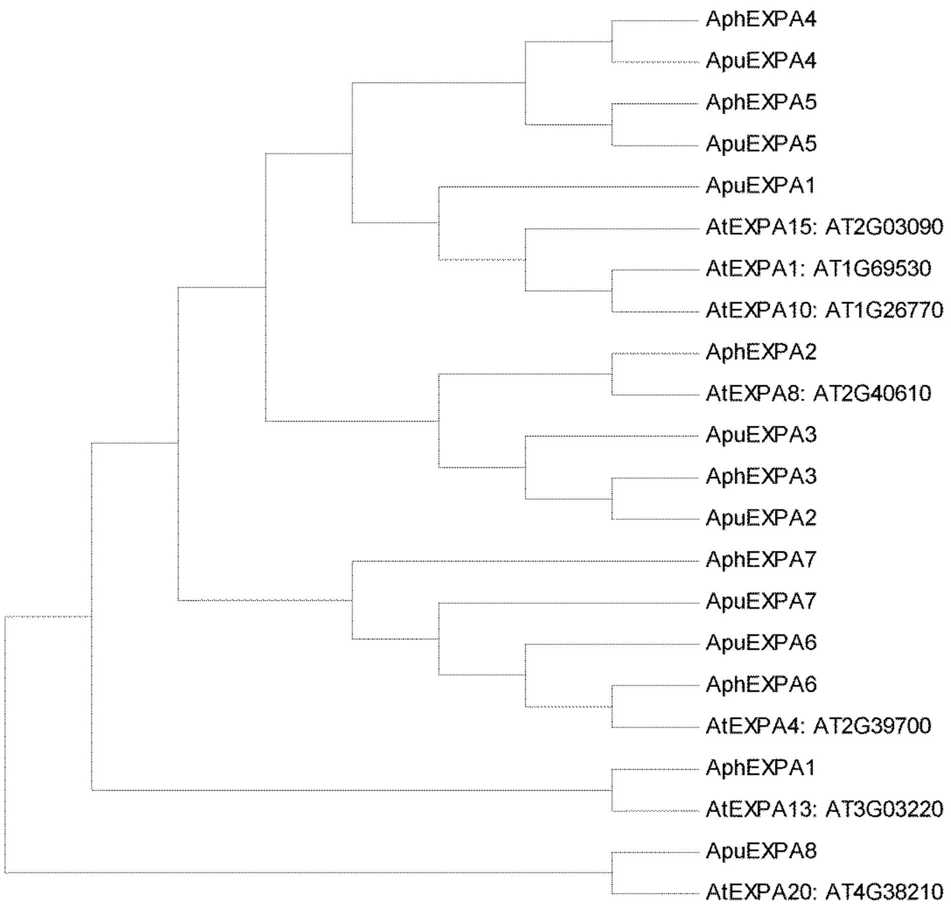

Supplement: Supplementary file 22 [file Image14.PDF]
