# Supplementary material for: Gene Expression Reaction Norms Unravel the Molecular and Cellular Processes Underpinning the Plastic Phenotypes of Alternanthera Philoxeroides in Contrasting Hydrological Conditions
Source: Front Plant Sci. 2015 Nov 12;6:991. doi: 10.3389/fpls.2015.00991 (PMC4641913; doi:10.3389/fpls.2015.00991)

Supplementary Figure 6. Temporal patterns of *A. philoxeroides* cell wall modification-related genes expression in contrasting hydrological habitats. The color bar represents the scale of the expression changes for each gene (log_2(pond/upland)). Only genes that exhibited different expression were shown. The corresponding numerical data are presented in **Supplementary Table 6**.

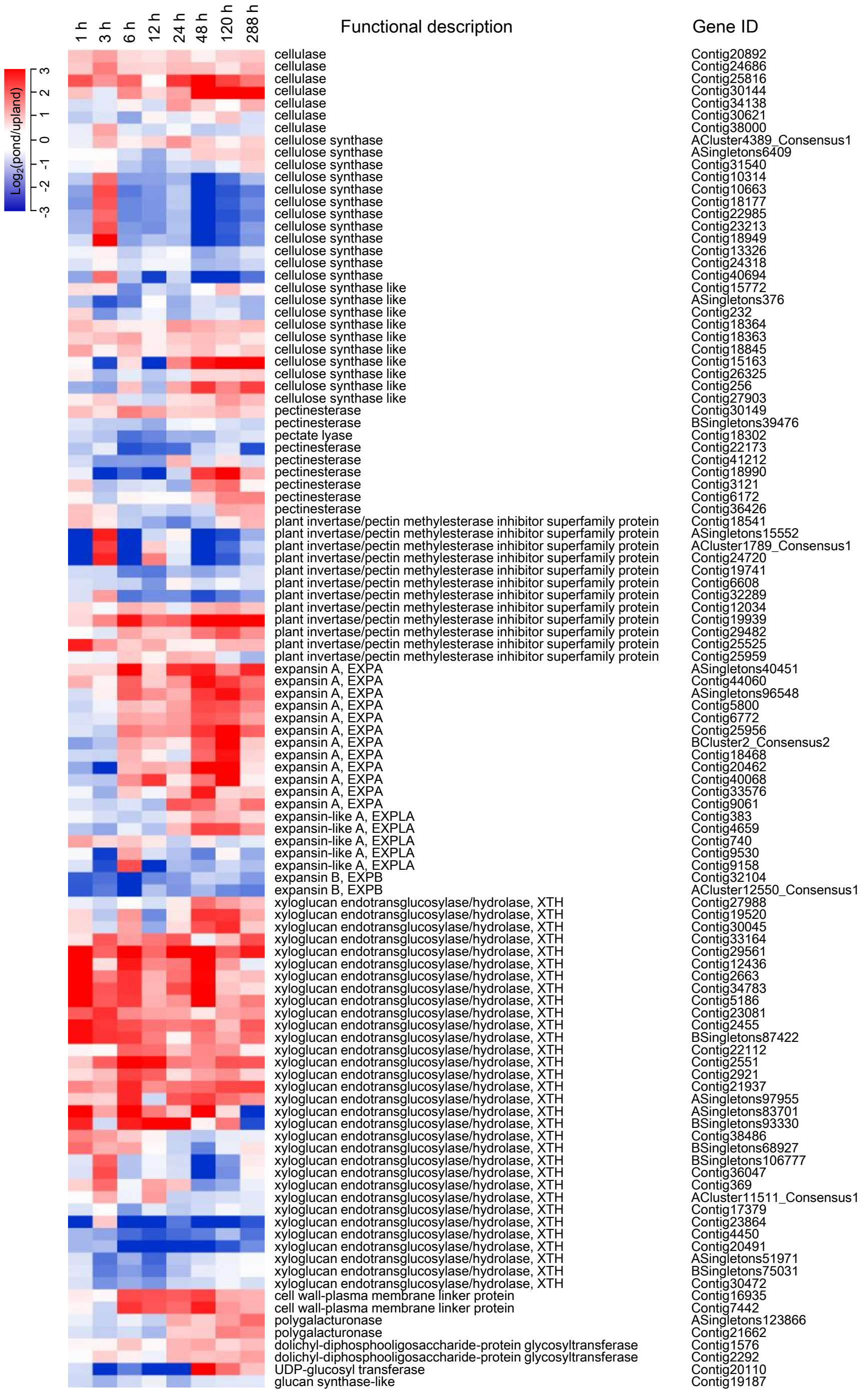

Supplement: Supplementary file 14 [file Image6.PDF]
